# Position of Carbonyl Group Affects Tribological Performance of Ester Friction Modifiers

**DOI:** 10.1021/acsami.3c16432

**Published:** 2024-03-08

**Authors:** Wei Song, Sophie Campen, Huw Shiel, Chiara Gattinoni, Jie Zhang, Janet S. S. Wong

**Affiliations:** † The Tribology Group, Department of Mechanical Engineering, Imperial College London, Exhibition Road, South Kensington, London SW7 2AZ, U.K.; ‡ Department of Material, Imperial College London, Exhibition Road, South Kensington, London SW7 2AZ, U.K.; § Department of Physics, King’s College London, Strand, London WC2R 2LS, U.K.

**Keywords:** friction modifiers, ester, isomers, tribochemistry, tribofilm, adsorption

## Abstract

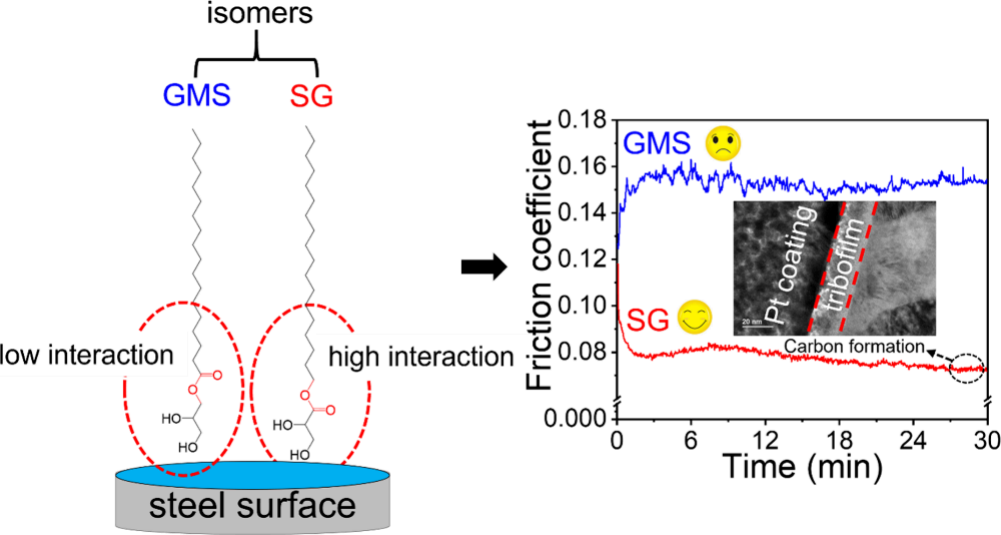

The tribological properties of lubricants can be effectively improved by the introduction of amphiphilic molecules, whose performance is largely affected by their polar head groups. In this work, the tribological performance in steel–steel contacts of two isomers, glycerol monostearate (GMS) and stearyl glycerate (SG), a glyceride and a glycerate, were investigated as organic friction modifiers (OFM) in hexadecane. SG exhibits a much lower friction coefficient and wear than GMS despite their similar structures. The same applies when comparing the performance of oleyl glycerate (OG) and its isomer, glycerol monooleate (GMO). Surface chemical analysis shows that SG forms a polar, carbon-based, tribofilm of around tens of nanometers thick, while GMS does not. This tribofilm shows low friction and robustness under nanotribology test, which may contribute to its superior performance at the macro-scale. The reason for this tribofilm formation can be due to the stronger adsorption of SG on the steel surface than that of GMS. The tribofilm formation can be stress-activated since lower friction and higher tribofilm coverage can be obtained under high load. This work offers insights into the lubrication mechanism of a novel OFM and provides strategies for OFM design.

## Introduction

Friction and wear cause energy and material loss in machinery.^1^ High-performance lubricant is required to resolve these issues.^2^ Friction modifiers (FMs) are commonly introduced into a base fluid to improve lubricity and energy efficiency, especially under the boundary lubrication regime where contact of rubbing surfaces occurs.^3,4^ The severe rubbing condition means that once friction modifiers reach rubbing contacts, they may be converted into a tribofilm of a different chemistry due to tribochemical reactions. Hence, the effectiveness of a FM usually depends on its interaction, as well as its reactivity with rubbing surfaces.^5^

**Figure 1:**
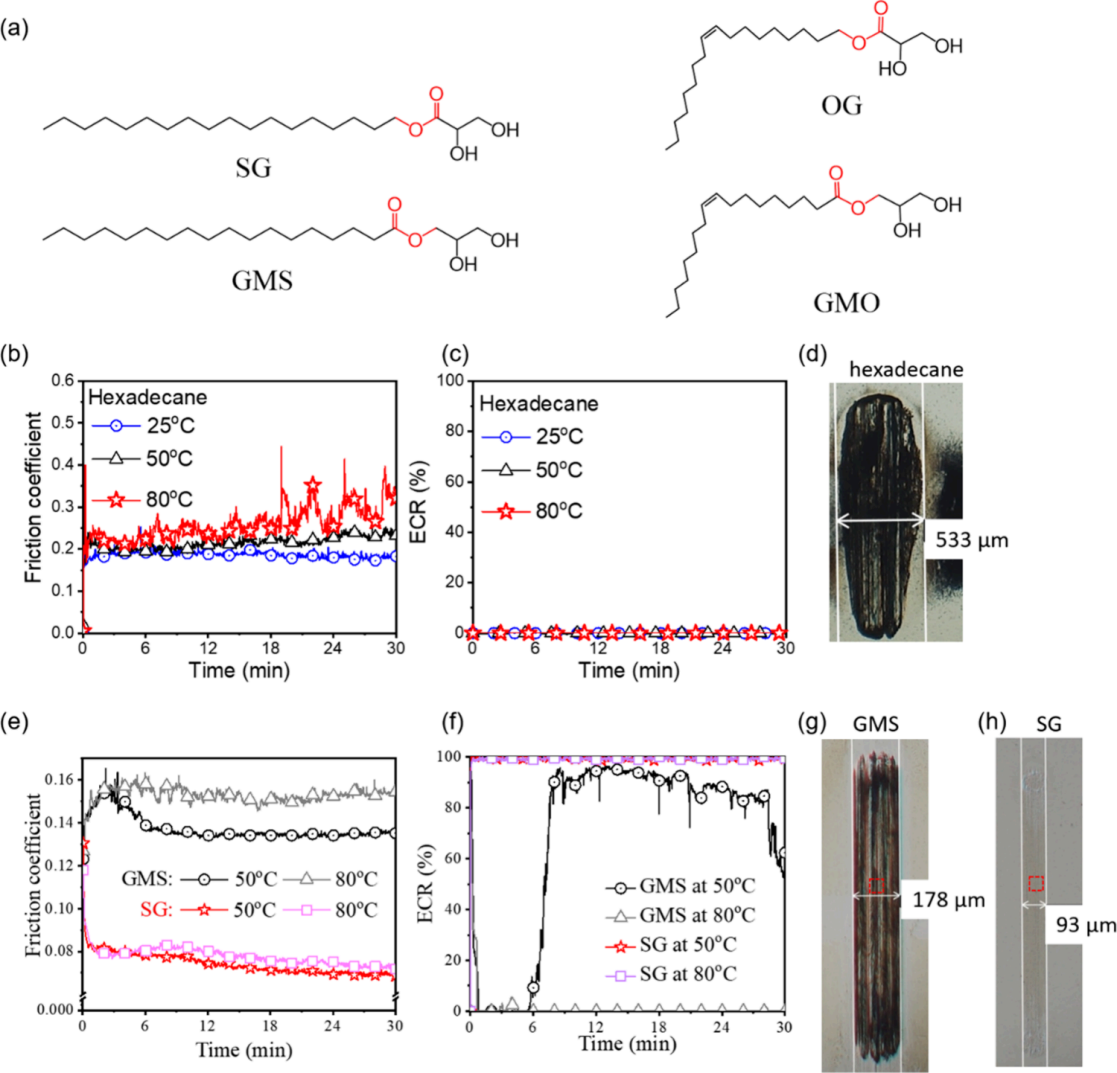
(a) Chemical structures of GMS, SG, GMO, and OG. For hexadecane: (b) friction coefficient and (c) ECR. For 1 mM GMS and SG: (e) friction coefficient and (f) ECR. Wear tracks on steel discs formed at 80 °C in (d) neat hexadecane, (g) 1 mM GMS, and (h) 1 mM SG. The red boxes in panels (g) and (h) are the areas where XPS was performed. All friction coefficients and ECR results are average of three tests. Images of wear tracks formed at 50 °C are in Figure S8 and are qualitatively similar. Panels (d,g,h) were taken before rinsing. Images of wear tracks before and after hexane rinsing are compared in Figure S9. Friction coefficients and ECR values for the first 6 min of the tests are in Figure S7.

Amphiphilic molecules, like oleic acid, glycerol monooleate (GMO), and glycerol monostearate (GMS), are composed of a nonpolar tail group and a polar headgroup, as shown in Figure 1a. GMO^6−8^ is a common organic friction modifier (OFM) whose tribological performance has been widely investigated and is utilized in commercial lubricant additive packages. It is believed to adsorb on metal surfaces by its polar headgroup through hydrogen bonding of one or more of the glyceryl hydroxyls to surface hydroxyls or oxygen atoms within an oxide layer on the metal surface. The ester carbonyl oxygen may also interact with iron centers on the iron oxide surface.^9^ It may then be hydrolyzed to oleic acid during rubbing by the small amount of water present on surfaces.^10^ Oleic acid, a proven friction modifier, generated by the hydrolysis of GMO, then forms a protective layer and effectively reduces the friction of the steel–steel contact.^11,12^ This proposed working mechanism of GMO is supported by some previous reports,^8,10,13^ which showed both GMO and oleic acid give similarly low friction coefficients. However, others have found contradictory evidence. For example, Koshima et al.^14^ reported that GMO exhibited a higher friction coefficient than oleic acid (0.132 vs 0.108) in sliding iron contacts at 100 °C. This casts doubt on the proposed mechanism of ester hydrolysis via tribochemical reaction. It has also been suggested that tribochemical reactions requiring water are unlikely to occur in hydrocarbon medium.^15^

To understand the working mechanism of GMO, we carry out a study using two different structural isomers of GMO where the direction of the ester group is reversed (Figure 1a). Hydrolysis of GMO yields oleic acid and glycerol, while hydrolysis of oleyl glycerate (OG) yields oleyl alcohol and glyceric acid. We expect that the observed friction response after hydrolysis will mainly be governed by the long-chain fatty moiety (oleic acid or oleyl alcohol). This is based on previous studies that showed lateral van der Waals forces between long alkyl groups to be important for the formation of stable low-friction boundary films.^16^ If the ester groups do undergo hydrolysis, we hypothesize that GMO will give lower friction than OG. This is because an alcohol headgroup in fatty alcohols has weaker interaction with steel surface than the carboxylic acid group in oleic acid and stearic acid.^17−19^ Hence, molecules such as oleyl alcohol and octadecanol are known to be poorer OFMs for steel–steel contacts.

Esters in themselves make relatively poor friction modifiers. Methyl oleate and methyl stearate gave significantly higher friction than their equivalent carboxylic acids.^20^ The two hydroxyl groups afforded by the glyceryl or glycerate headgroup are of paramount importance for initial adsorption of GMO and OG at the metal surface. Thus, OG could give lower friction than oleyl alcohol. If hydrolysis occurs during rubbing, we may then see an increase in friction with time as the oleyl glycerate is converted to oleyl alcohol. Note that the description so far ignores the contribution of glycerol and glyceric acid to the boundary film, which may affect the overall performance of the OFM boundary film. In the case that the ester groups do not undergo hydrolysis but instead remain intact, we hypothesize that the observed friction coefficient will be similar for both GMO and OG. Note that GMO and GMS may also undergo tribochemical reactions other than hydrolysis.^21,22^ In this case, identification of the resulting products will shed light on the working mechanisms of these OFMs.

While the principal goal of this study is to better understand the behavior of GMO, we simplify our investigation by researching its saturated analogue, GMS. It is well-known that the cis conformation of the C=C double bond in the oleyl group causes the hydrocarbon chain to adopt a bent structure, which means it is unable to form a close-packed monolayer.^23^ As a result, oleic acid tends to exhibit higher boundary friction in steel contacts than the saturated straight chain stearic acid.^24^ Using GMS allows us to focus on the role of the glyceryl or glycerate ester headgroup. This also avoids any potential complications caused by having an additional reactive center in the middle of the hydrocarbon chains.^8^ The main research question is thus: how does the tribological performance of stearyl glycerate (SG), an esterification product of octadecanol and glyceric acid, compare to that of GMS? And importantly, what does this tell us about the working mechanism of these additives?

## Results

### Tribological Performance of FMs

This paper focuses on the tribological performance of SG and GMS, a glycerate, and a glyceride. Performance of OG and GMO has also been examined (see Supplementary Note 2) and is qualitatively similar to SG and GMS, respectively. This suggests that the structure of the tail groups of the OFMs has a minimal effect on our observations, and the difference in friction coefficients between GMS and SG (GMO and OG) is attributed to the difference in head groups. Tests have been conducted with 1 and 10 mM OFM solutions, giving similar results (Figure S11). Hence, only results from 1 mM solutions are discussed below.

Figure 1 shows the friction coefficients of hexadecane with and without OFMs in steel–steel contacts and their corresponding electrical contact resistance (ECR) values. Note that high ECR values suggest complete separation between the rubbing surfaces by nonconducting films, which is the case for OFM monolayers. Friction coefficient obtained with neat hexadecane is stable and is around 0.20 at 25 °C (Figure 1b). It increases slightly with time at 50 °C. At 80 °C, it increases further and rises more chaotically with time. This is indicative of wear, see images of the steel wear track (Figure 1d). At all temperatures, the ECR is zero (Figure 1c), indicating substantial metal–metal contacts in neat hexadecane.

The friction coefficient with 1 mM GMS at 50 °C increases, reaching a maximum of 0.15 at rubbing time *t* = 3 min, while ECR remains zero. Friction then reduces and stabilizes at a lower value of 0.14. This coincides with an increase of ECR to 80–90% (circles, Figure 1f), suggesting the formation of a tribofilm. At 80 °C, ECR is always 0 (triangles), with friction coefficient stable and remaining high at ∼0.15.

The friction coefficient with 1 mM SG at 50 °C initially is ∼0.13 initially. It then decreases quickly to a much lower value of ∼0.08 (stars, Figure 1e) while ECR value increases to 100% in less than 1 min (Figure 1f). This suggests that a nonconductive, friction reducing tribofilm forms rapidly during rubbing. The test conducted at 80 °C gives a similar result. Wear tracks formed in SG (Figure 1h) are smoother and narrower than those formed in GMS (Figure 1g), showing that the SG tribofilm reduces wear as well as friction.

Our results show that both glycerate and glyceride reduce friction and wear of steel–steel contacts in hexadecane. The two glycerates, OG and SG, however, give lower friction and provide better wear protection than the glycerides GMO and GMS. This is due to the formation of a tribofilm when glycerate is used. Our results are relatively insensitive to the range of temperature tested and tail group structures. A summary of friction results is in Figure S10.

To examine the stability of the glycerate tribofilm, a 30 min friction test was conducted in 1 mM OG where the test was paused, and the ball lifted away from the disc intermittently at time = 2 and 4 min. The friction coefficient remains at 0.07 despite the pauses, as shown in Figure S12. This suggests that the glycerate tribofilm is stable once it is formed. This allows the tribofilm to undergo further ex situ examination.

### Surface Morphology of Wear Tracks

**Figure 2:**
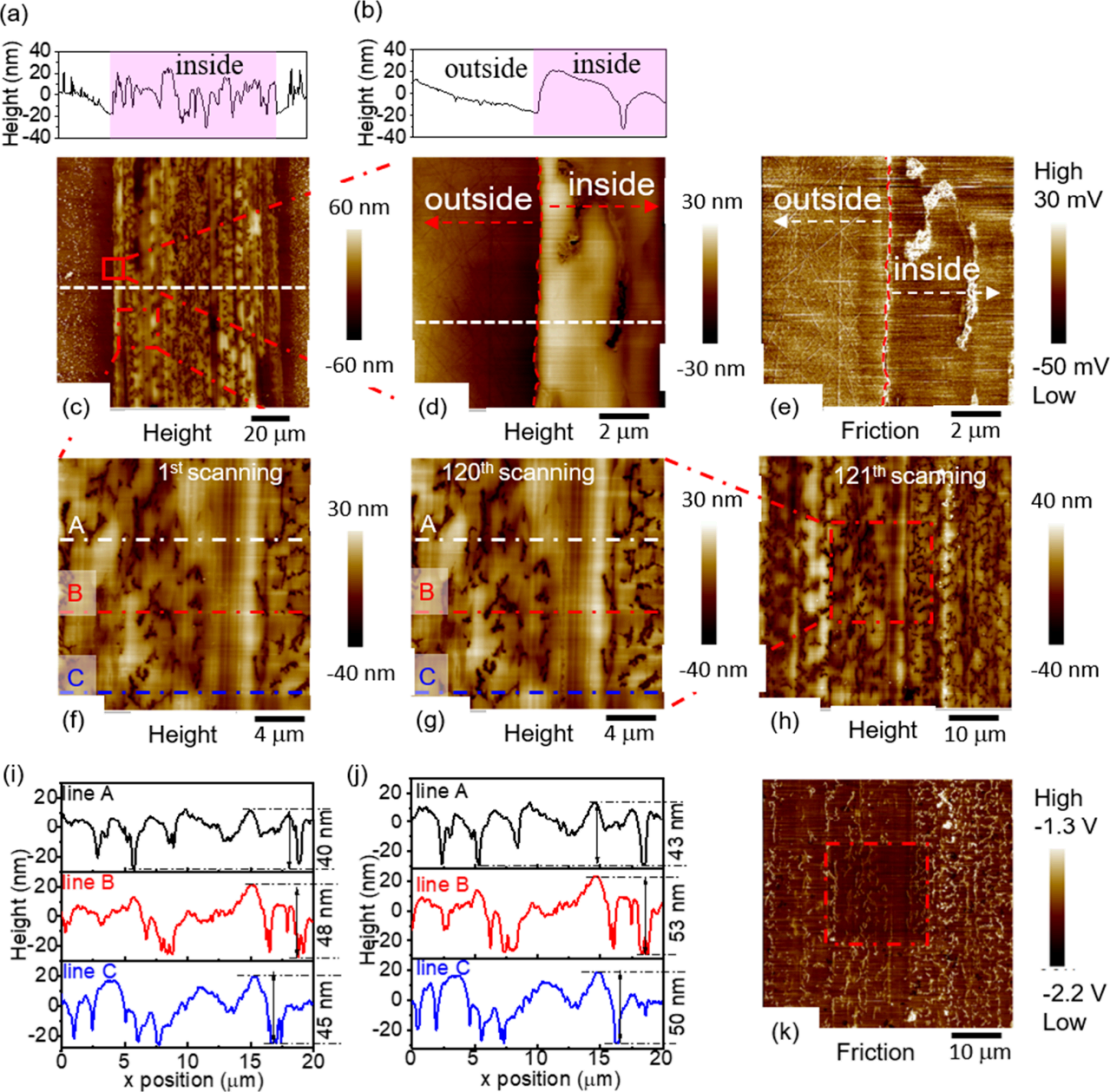
Morphology of steel disc wear track formed in 1 mM SG at 80 °C. (a, b) The height profiles of the white lines in panels (c) and (d), respectively. (c) AFM height image of the wear track. (d) A magnified image of a region near the edge of the wear track, see location in panel (c). (e) The corresponding lateral force image of (d). (f) Height image of another region in panel (c). (g) Height image of the same region in panel (f) after 120 scans. (i, j) Line profiles in locations, see lines A, B, and C in (f) and (g), respectively. (h) Height and (k) lateral force images of the area in panel (g) and its surroundings.

**Figure 3:**
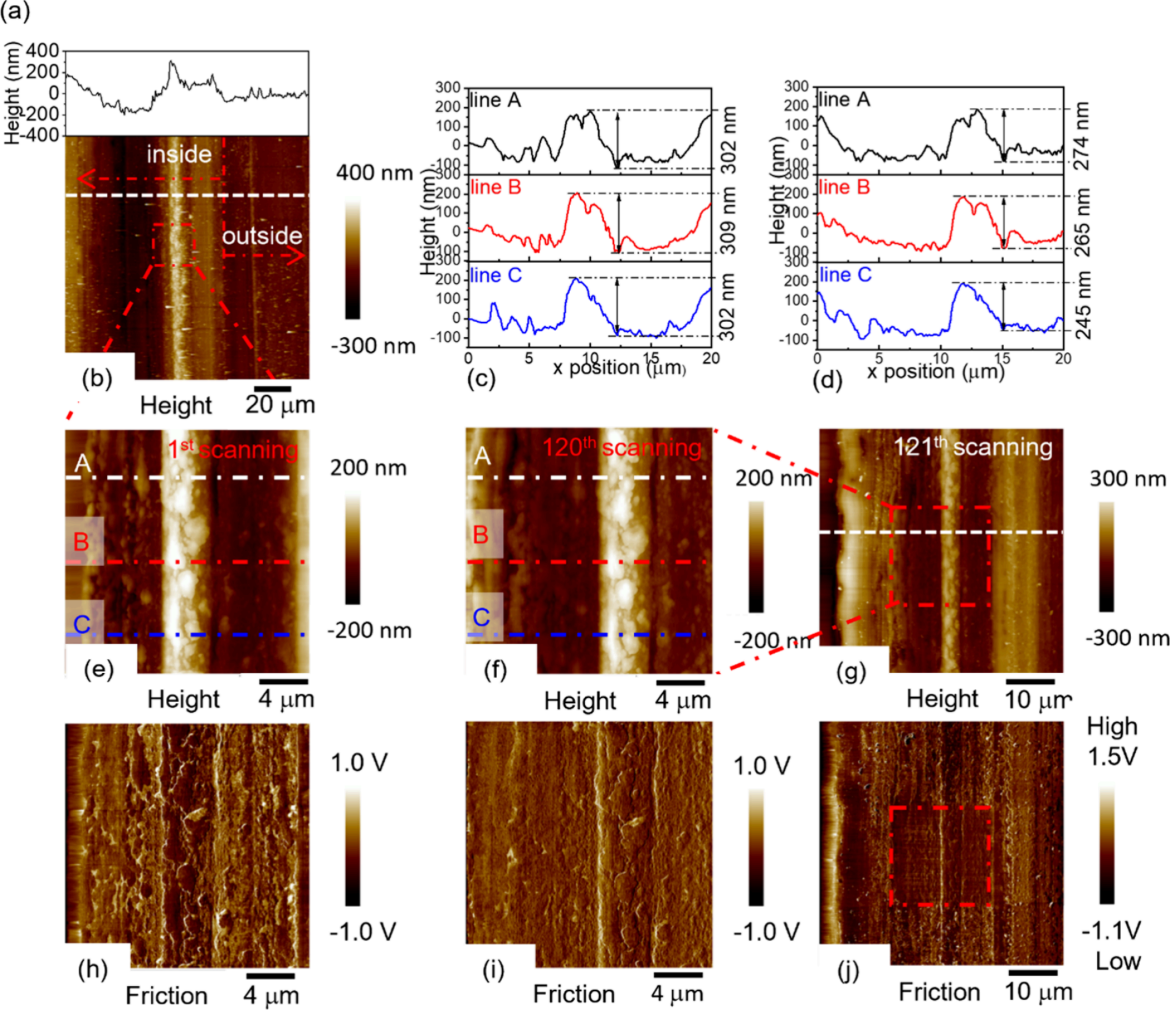
Morphology of steel wear track formed in 1 mM GMS in hexadecane at 80 °C. (a) The height profiles of the white line in (b) AFM topography of the wear track. The red dashed line indicates the edge of the wear track, with left and right of the line being inside and outside the track. (c, d) The height profiles of lines A, B, and C in panels (e) and (f). (e) AFM topography of the red box region in panel (b). (f) AFM topography of panel (e) after being scanned by the AFM tip for 120 times. (g) Topography of panel (f) and its surrounding. (h–j) The lateral force images of panels (e), (f), and (g) respectively.

Wear tracks on steel discs formed in 1 mM SG and GMS at 80 °C were examined by AFM using a Si_3_N_4_ tip in a flow cell filled with hexadecane, see Figures 2 and 3.

The wear track formed at 80 °C in 1 mM SG is covered by features that looked like a cracked film (Figure 2). Besides cracks, the film is relatively continuous and appears smooth when imaged at a small scan size (Figure 2d). This tribofilm had a maximum thickness of about 50 nm (Figure 2a,b).

Compared to a bare steel disc surface (Figure S13), the SG tribofilm completely covers the polishing marks on the steel disc and gives a lower friction (Figure 2e). The tribofilm formed by OG has similar characteristics, see Figure S14.

The durability of the SG tribofilm is evaluated by scratching it with an AFM tip 120 times under contact mode. Assuming a slightly worn tip with a radius of 100 nm, the mean contact pressure was about 1.3 GPa. Images taken during the first and the 120th scans (Figure 2f,g) and their accompanied line profiles (Figure 2i,j) show that the morphology of the film is hardly affected by scratching. The friction of the scratched region (inside the square, Figure 2k) is, however, more uniform than its surrounding tribofilm (see also Figure S15). This suggests that some materials on the very top layer of the tribofilm may have been redistributed to the originally uncovered part of the surface during repeated scratching, resulting in a more uniform friction distribution. Overall, this tribofilm is strong and can protect the steel surface from wear while reducing friction.

The wear track formed in 1 mM GMS at 80 °C is a groove with a width of about 100 μm and a depth of about 100 nm, see Figure S16. Most of the wear track shows little sign of tribofilm; however, differentiation between tribofilm and the steel surface is not straightforward owing to the large amount of wear and the increased roughness of the rubbed surface. A thin strip of patchy, low friction film can be seen close to the inner edge of the wear track (Figure 3b,e). This film, unlike the SG tribofilm, appears to contain discrete features or particles of approximately 5 μm in size. The height of these features decreases slightly after they are scratched by the AFM tip 120 times (compare Figure 3c,d). This is accompanied by a homogenization of friction in the scratched area, likely due to the redistribution of scrapped materials (Figure 3i,j). This suggests that GMS generates a relatively weak, patchy, low friction film on steel, which are found in small areas of the wear track after the test. Previously, molecular dynamics simulations suggested that GMO adsorbs onto ferrous surfaces as reverse micelles, which then disintegrate under shear.^25^ Thus, the observed patchy film could be due to adsorbed reverse micelles of GMS that have been impacted by shear, perhaps partly coalescing.

### Chemical Analysis of Wear Tracks

**Figure 4:**
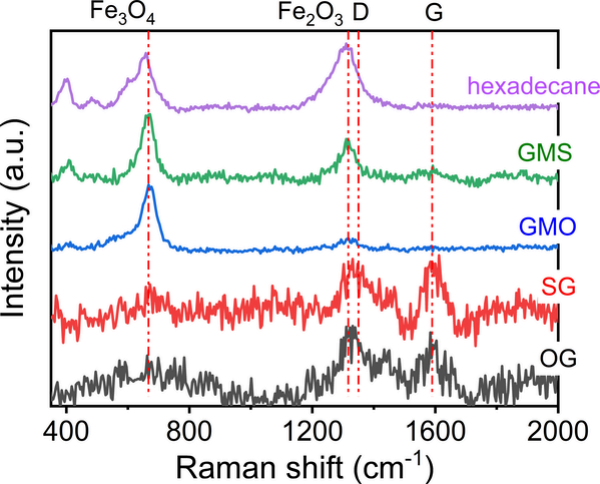
Background-corrected and normalized Raman spectra of wear tracks formed at 80 °C in hexadecane with and without 1 mM additives. The intensity of the strongest peak of each spectrum is set at 1. Raw spectra are shown in Figure S17. Raman spectra of wear tracks formed in SG and OG at other temperatures are in Figure S18.

Wear tracks formed in all tested lubricants were examined with Raman spectroscopy, see Figure 4.

The Raman spectra of wear tracks formed in neat hexadecane and 1 mM GMS show strong peaks at 667 and 1317 cm^–1^, which are assigned to Fe_3_O_4_^26^ and Fe_2_O_3_,^26^ respectively. Note that the D band of amorphous carbon,^27−29^ which is around 1350 cm^–1^, and the Raman peak of Fe_2_O_3_ are at proximity. So, some carbonaceous materials may be on these surfaces.

The spectrum of the wear track formed in 1 mM SG is noisy due to its low intensity. It contains two peaks at 1350 and 1580 cm^–1^ that are attributed to the D band and G band and suggest the existence of carbon-based materials. The intensities of these carbon peaks are however very weak, indicating that the film is very thin. Their intensities may also depend on severity of the rubbing. Note that in this case, the intensity of the G-band is reduced with laser exposure. G-band is sometimes denoted to graphitic carbon,^30,31^ which may contribute to low friction.^32^ Further tests are necessary to pinpoint the chemistry of the film. Importantly, no Fe_3_O_4_ peak is seen, which supports the idea that the glycerate (SG) tribofilm protects steel rubbing surfaces. Spectra obtained from wear tracks formed in 1 mM GMO and OG are similar to those in 1 mM GMS and SG, respectively.

X-ray photoelectron spectroscopy (XPS) reveals that the surface of an unrubbed area is composed of Fe and O with a small amount of C in the form of C–Fe,^33,34^ suggestive of a thin layer of iron oxide on steel (see Figures S19 and S20a), while top surfaces of wear tracks formed in 1 mM SG and 1 mM GMS have higher C concentration and lower Fe concentration than the unrubbed area (Figure S19).

**Figure 5:**
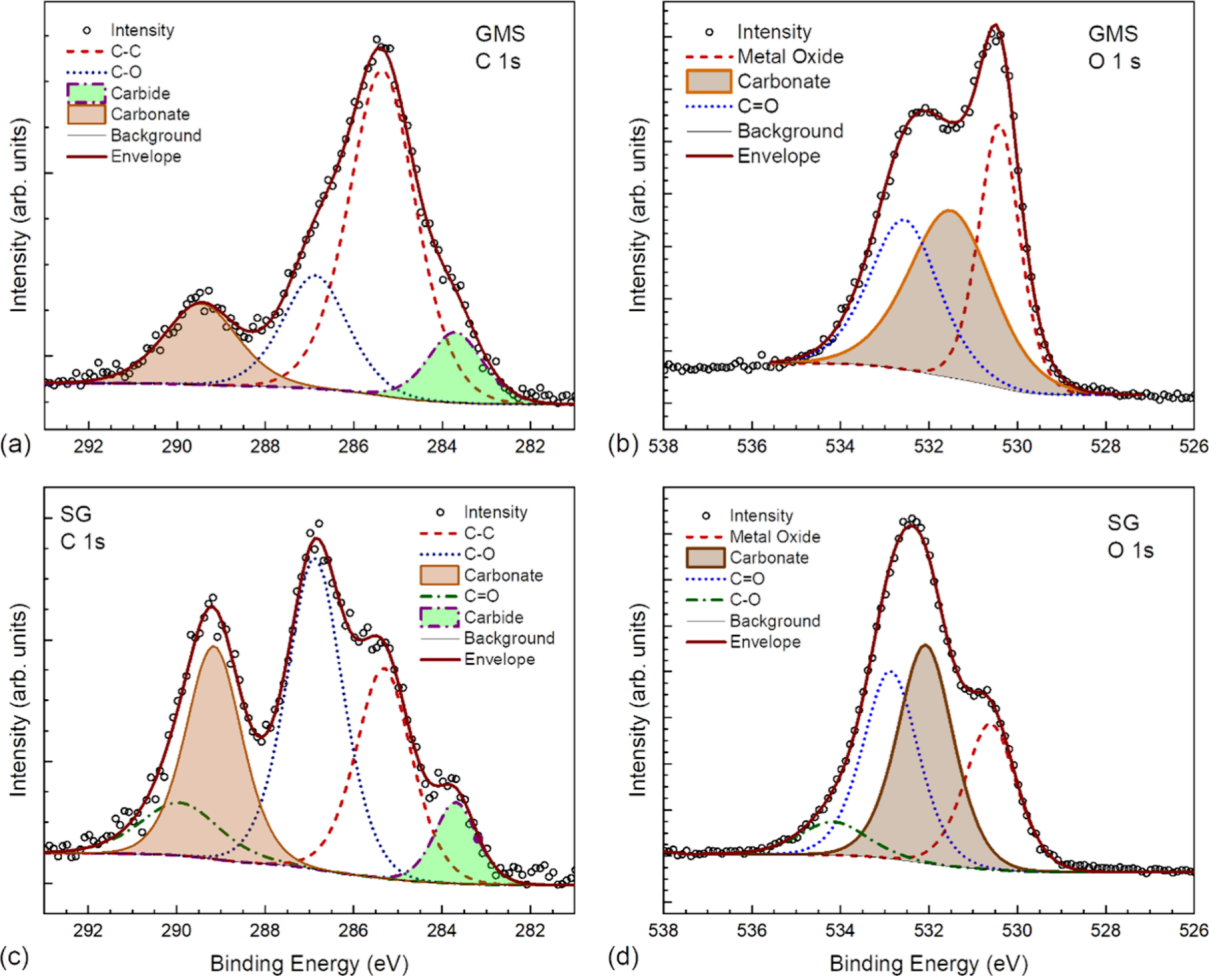
(a, c) C 1s and (b, d) O 1s XPS high resolution spectra of 1 mM GMS lubricated and 1 mM SG lubricated surfaces, respectively.

The top surface of the wear track formed in 1 mM GMS contains carbon compounds, mainly composed of −C–C–, some polar carbon^35^ (−C–O−), and carbonate moieties, as well as iron oxide, which may come from wear debris (Figure 5a,b). Comparing the surface compositions of SG and GMS tribofilms reveals that SG tribofilms contain more polar carbon species (−C–O– and −O–C=O−) and carbonate moieties (Figure 5c). This may lead to a stronger adhesion between the SG tribofilm and the steel substrate. A lesser proportion of metal oxide species to polar carbon species is seen in SG films, confirming results from Raman spectroscopy. Note that iron carbide is likely from bulk steel. Combined with their weak D- and G-bands in Raman spectra, it suggests that these surfaces only have very little amorphous or graphitic carbon materials.

Results from ToF-SIMS, presented in Figures S21 and S23, show that the GMS film consists of more lower molecular weight constituents than the SG tribofilm. Alternatively, the results may mean the GMS film can be more easily broken down into lower molecular weight fragments during ToF-SIMS.^36^ This supports the idea that the GMS tribofilm is relatively weak. The higher molecular weight fragments seen in the SG tribofilm, on the other hand, together with their polar nature may explain why SG is able to form a robust tribofilm. Note that ToF-SIMS found more iron oxide fragments (FeO_2_^–^) on the GMS lubricated surface, which is consistent with results from Raman and XPS.

**Figure 6:**
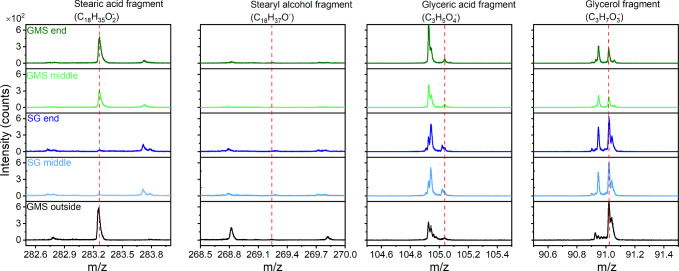
ToF-SIMS spectra of worn surfaces, focusing on regions of potential hydrolyzed products.

ToF-SIMS (Figure 6) reveals ions that may be assigned to stearic acid (C_18_H_35_O_2_^–^) and glycerol (C_3_H_7_O_3_^–^) on the worn surface formed in 1 mM GMS, which supports the hydrolysis of GMS as suggested by the literature. Note that the molecular ion of GMS is not observed. Interestingly, only a very small amount of glyceric acid (C_3_H_5_O_4_^–^) and no stearyl alcohol are found on worn surfaces formed in 1 mM SG. This suggests that SG may not have decomposed to stearyl alcohol and glyceric acid via hydrolysis. Rather, it has followed a different reaction path that gives rise to a highly oxidized film, evidenced by multiple C_*x*_H_*y*_O_*z*_ peaks in ToF-SIMs (see Figures S21 and S22). This tribochemical reaction can lead to compounds with higher molecular weight than pristine SG.^21^

### Growth of SG Tribofilm

**Figure 7:**
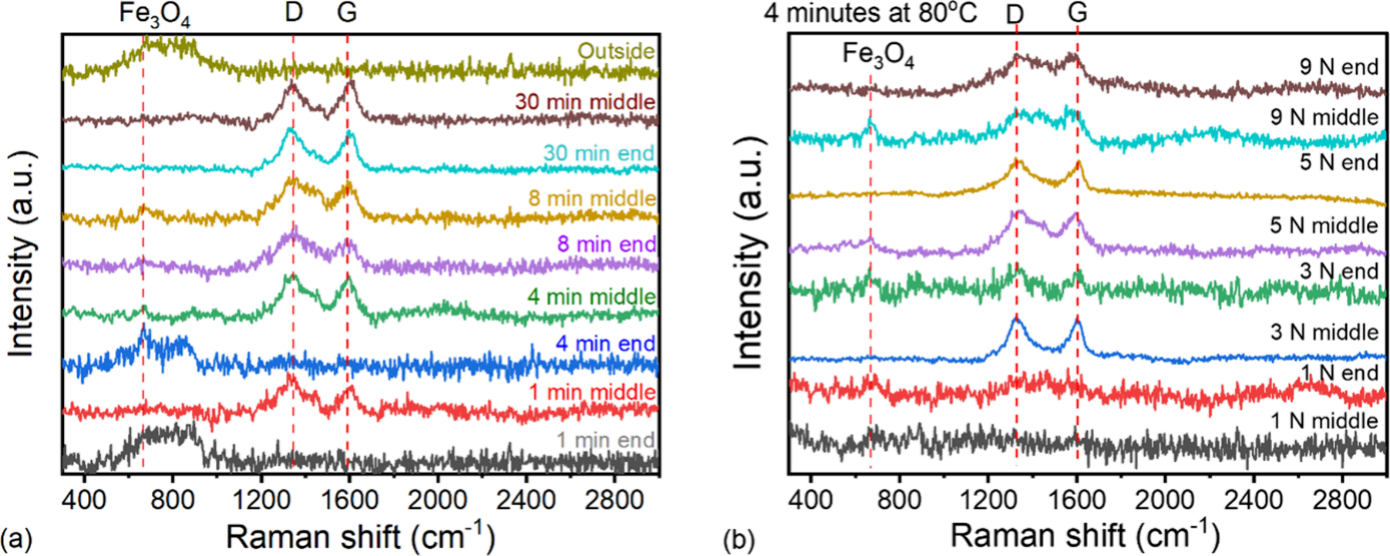
Raman spectra of wear tracks on steel discs formed in 1 mM SG. Spectra were collected at positions corresponding to the midstroke and end of the stroke motion of rubbing. (a) Load = 5 N and durations of test were set at 1, 4, 8, and 30 min; (b) duration of test = 4 min and load = 1, 3, 5, and 9 N.

**Figure 8:**
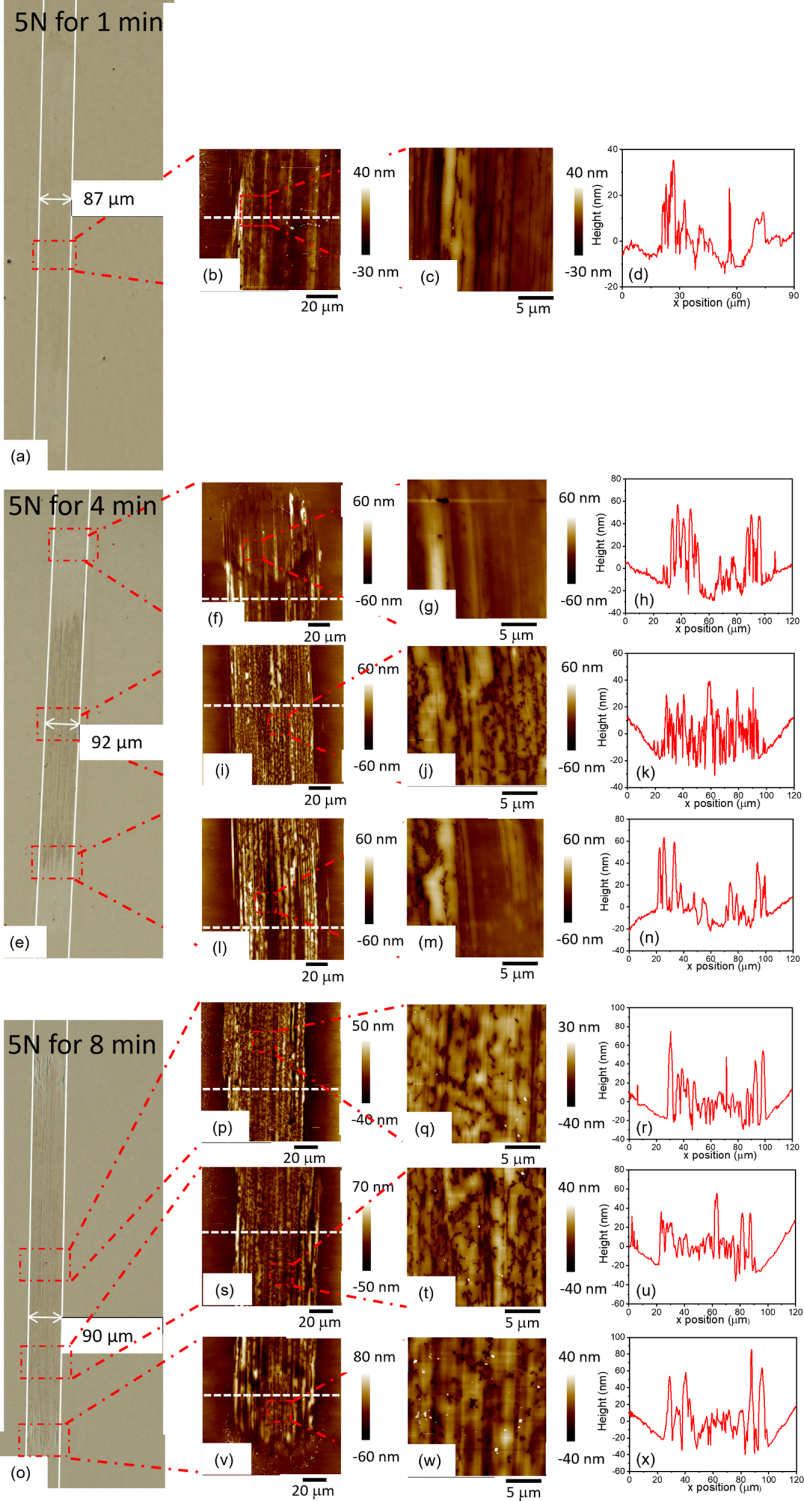
Morphology of wear tracks formed in 1 mM SG at 5 N with different durations. Optical micrographs, AFM height images, and corresponding line profiles for (a–d) 1 min, (e–n) 4 min, (o–x) 8 min: (a, e, o) optical images; (b, c, f–m, p–w) AFM topographic images; (d, h–n, r–x) height profile of the white dashed line in the AFM images on the same row.

The formation of SG tribofilm was investigated with rubbing tests of durations equal to 1, 4, 8, and 30 min and an applied load fixed at 5 N, see Figure S25. At a duration set at 4 min, tests were also conducted at different loads of 1, 3, 5, and 9 N to investigate the effect of contact pressure on the initiation of tribofilm growth, see Figure S27. Raman spectra (Figure 7) were taken at locations corresponding to the midstroke and end-stroke positions on the wear tracks. Morphology of wear tracks was observed with optical microscopy and AFM (Figure 8).

When load = 5 N, Raman signals from D- and G-bands can be obtained near the midstroke position of the track after 1 min of rubbing. AFM images (Figure 8a) reveal that the SG tribofilm at this stage is more prominent at the edge of the track, where contact pressure is low. As time progresses, the thickness of the tribofilm, as well as its coverage on the wear track, increases (Figure 8e and S26). After 8 min of rubbing, a tribofilm has developed throughout the whole track, see Figures 7a and 8o. This coincides with a low and stable friction coefficient. When carbon peaks are not observed, weak iron oxide peaks are revealed, and note that these normalized spectra are noisy due to low peak intensities.

When the test duration is fixed at 4 min, low friction is achieved only if load ≥3 N (see Figure S27). Raman spectra confirm that carbon-based tribofilm is formed at load ≥3 N and it builds up at midstroke position first. At load = 9 N, the D- and G-peaks from the tribofilm are observed throughout the whole wear track, suggesting its film formed and stabilized the quickest. Steady-state shear stresses are similar at load ≥3 N (see Figure S28), suggesting that the nature of the steady state tribofilm remains the same even though high load results in thinner film. On the other hand, the maximum contact shear stress occurs during the early stage of rubbing and increases with load from 3 to 9 N. This suggests high stress promotes film formation, although it is difficult to deconvolute the effects of compression and shear stresses. Interestingly, a tribofilm does begin to form at 1 N; however, it only covers a small area of the rubbing surface (Figure S30), thus perhaps is not extensive enough to lower the macroscale friction. A 10 h test at 1 N was also conducted, and it shows that an extensive tribofilm does eventually form. However, this film has a different morphology (it is not elongated in the sliding direction, as observed by AFM) and chemistry (it does not exhibit the G-band by Raman spectroscopy; Figure S29). This implies that a critical compressive stress exists for formation of the low-friction tribofilm.

## Discussion

GMS and SG, glycerides, and glycerates are isomers. Yet, only SG forms a robust, polar, carbon-based tribofilm during rubbing. The formation of the tribofilm is not spontaneous, but the film eventually covers the whole rubbing surface and results in a low friction. This tribofilm is tens of nanometers thick and resists scratching by an AFM tip. For GMS, patches of the tribofilm can be seen mostly near the inner edge of the wear track. This film is relatively weak and can be redisturbed by an AFM tip. Raman spectra of SG tribofilm reveal D and G-bands, and XPS shows that it contains more polar compounds with −C–C–, −C–O–, and O–C=O groups, while results from ToF-SIMS show that it also contains carbon compounds with higher molecular weight than GMS tribofilm.

It is commonly believed that the formation of SG and GMS tribofilms involves the dissociation of these molecules via ester hydrolysis. While ToF-SIMS shows stearic acid on GMS wear tracks, supporting the idea that GMS tribofilm may have been produced via ester hydrolysis, no stearyl alcohol was found on SG wear tracks. This suggests that either ester hydrolysis is not involved in the formation of SG tribofilm or it only produces intermediates, which are further oxidized to form the SG tribofilm.

QCM results show that the OG adsorbed film has higher mass than GMO on iron oxide surfaces after surface excess mass has been removed by flushing with hexadecane (Figure S33). The surface density of OG is calculated to be about 1.22 molecules per nm^2^, which is above the threshold surface coverage of good organic friction modifiers.^6^ It is expected that an adsorbed SG film will have higher surface coverage than an OG film due to SG having a straight chain tail.^6^

If these friction modifier molecules are surface-adsorbed prior to hydrolysis, then nucleophile attack at the carbonyl carbon center may be more sterically hindered than typically encountered in solution. So, the difference in degradation paths taken by GMS and SG may stem from the conformation of their ester groups when adsorbed at the surface or alternatively from the different surface activities of their hydrolysis products. Upon hydrolysis of SG, glyceric acid may adsorb preferentially to stearyl alcohol, yielding a highly oxidized and reactive OH-terminated film, which undergoes subsequent (tribochemical) condensation reactions to form a low friction carbonaceous film.

The SG tribofilm has more polar species than the GMS tribofilm as shown by ToF-SIMS. The observations of D- and G-bands in the Raman spectra of SG tribofilm, although very weak, suggest that this tribofilm is carbon-based. These bands are not seen in the spectrum of the GMS tribofilm. Note that there is now increasing evidence that a carbon-based tribofilm can form in a rubbing contact even with neat hydrocarbon base fluids^37,38^ and vapor.^22^ Thus, the carbon-based SG film may be from the degradation of hexadecane or SG, which may be facilitated by frictional heating. The average contact temperature rises due to frictional heating, estimated based on the Jaegers model,^39^ is low in our cases (see Table S2). The degradation of OFM may however still occur at asperity–asperity contacts where temperature can be substantially higher.^40−43^ Reactions can also be facilitated by shear stresses,^37,44^ promoting the formation of tribofilm locally via a mechanochemical route. This is supported by our observation that a critical load is necessary for the SG tribofilm to form and low friction coefficient to be achieved (Figure 8). At higher load, the tribofilm is formed more quickly even though a similar shear stress remains (Figures S30–S32). This implies that increasing the load above the critical threshold (from 3 to 9 N) affects the formation rate but not the chemistry of the film. The formation of the tribofilm in the steel–steel contact is thus at least partially mechanically activated.

## Conclusions

OFMs are amphiphilic molecules that are added to lubricants to reduce friction. In this work, the performance of two OFM isomers, GMS and SG, is investigated. GMS, a glyceride, is expected to undergo hydrolysis and form a carboxylic acid during rubbing, which according to the literature then interacts strongly with steel and forms a low friction film. SG is a glycerate. Should SG undergo hydrolysis, it is expected to form a fatty alcohol, which has been shown previously to weakly interact with steel in hexadecane. GMO and OG are also used, and they give similar results to GMS and SG, respectively.

Our results support the notion that GMS forms a tribofilm via ester hydrolysis. Some GMS tribofilms can be seen mainly near the inner edge of the wear track. This film is, however, relatively weak and can be redisturbed by an AFM tip. For the SG tribofilm, our results suggest that ester hydrolysis is either not involved or is only an intermediate step. This SG tribofilm is robust and is very different from conventional OFM, monolayer-type film. It reduces friction and offers protection to the rubbing surfaces from wear. The formation of the SG tribofilm in the steel–steel contact is at least partly mechanically activated. Surface chemical analysis suggests that this robust tribofilm contains more polar carbon-based materials and is of higher molecular weight than the GMS tribofilm. Both the existence of more polar compounds and higher molecular-weight fragments lead to a more adherent and stronger tribofilm against shear.

The result shows that despite GMS and SG being isomers, they undergo very different tribofilm formation mechanisms. This is likely due to difference in their adsorption and its adsorbed molecular conformation on the surface. The stronger adsorption and more protected carbonyl carbon center of adsorbed SG give rise to a polar, carbon-based film that is surprisingly strong and gives low friction. Future studies will be required to determine the glycerate tribofilm formation mechanism.

Organic friction modifiers play a very important role in the use of greener and lower viscosity lubricants. While a lot of attention has been invested in GMO, our work shows that glycerates can give better tribological performance. Apart from low friction, they also protect surfaces from wear. This is a path that is largely unexplored in the literature and should be taken into consideration when designing OFMs.

## Experiment

### Materials

The hexadecane (99%), glycerol monoostearate (GMS, ≥ 99%), glyceryl monooleate (GMO, ≥ 99%), aluminum oxide (activated, neutral) molecular sieves (3 Å), silica gel (Davisil grade 633, 200–525 particle size), and hexane (anhydrous, 95%) were all purchased from Sigma-Aldrich. Hexadecane was filtered through activated alumina, molecular sieves, and silica gel prior to use. Stearyl glycerate (SG) and oleyl glycerate (OG) were synthesized by the method in the previous literature,^45^ and their purity was confirmed by ^1^H NMR and ^13^C NMR, as shown in Supplementary Note 1. One millimolar SG (0.0463 wt %), 1 mM GMS (0.0463 wt %), 1 mM OG (0.0461 wt %), and 1 mM GMO (0.0461 wt %) in hexadecane were prepared by stirring for 30 min. Note that GMS and SG cannot be dissolved in hexadecane at room temperature, so that these two FMs were heated at 50 °C in hexadecane with stirring to form the solution.

### Tribological Tests

Tribological tests were carried out on a high-frequency reciprocating rig (HFRR, PCS instrument) with a ball-on disc geometry. Both balls and discs, provided by the PCS instrument, are composed of steel 52100 with roughness around 15 nm. Their Young’s modulus is 207 GPa, while the Possion’s ratio is 0.3. The diameter of the balls is 6 mm. The diameter of discs is 10 mm with a thickness of 3 mm. Prior to a tribological test, all balls and discs were cleaned with toluene under ultrasonication for 30 min, followed by rinsing with 2-propanol and drying with a high-pressure air gun. After that, they were cleaned in oxygen plasma for 2 min. The ball and discs were then mounted on the HFRR holder. The disc was heated to 50 °C before warmed SG/GMS solution was added. This is to avoid SG/GMS precipitation on the disc surface. The solution was warmed to the test temperature for 2 min before the friction test.

Reciprocating friction tests were performed at 100 Hz under 5 N normal load with a stroke length of 1 mm. The friction tests of hexadecane containing OG and GMO were carried out at 25, 50, and 80 °C. Hexadecane containing SG and GMS was tested at 50 and 80 °C because SG and GMS cannot be dissolved in hexadecane at 25 °C. Lubricant film thicknesses were calculated by Dowson and Hamrock’s Equation,^46^ and they were 8 nm at 25 °C, 5.7 nm at 50 °C, and 4.4 nm at 80 °C. Thus, the ratio of lubricant film thickness to surface roughness were all lower than 1, suggesting that all tests were carried out in the boundary lubrication regime. During rubbing, the electrical resistance (ECR) across the two rubbing surfaces were recorded, with 0 and 100% signifying intimate and no contact between the two surfaces, respectively. This is an effective way to monitor tribofilm formation during the friction test.^47^ All of the friction tests were run three times, and they gave good reproducibility. The discs after the friction test were rinsed with hexane for further analysis.

### Characterization

The ^1^H and ^13^C nuclear magnetic resonance (NMR) patterns of SG and OG were acquired on a Jeol 400 MHz spectrometer. Dimethyl sulfoxide (DMSO-*d*_6_) was employed as a solvent with tetramethylsilane (TMS) as the internal standard.

The topography of the wear track was investigated by atomic force microscopy (AFM, Bruker Multimode AFM with Nanoscope V controller) under tapping mode, while the lateral force on wear track surface was measured under contact mode with 1.5 V deflection set point. To assess the strength of tribofilm, an AFM tip was used to scratch the tribofilm surface at 10 V deflection set point for 120 times. A triangular Si_3_N_4_ cantilever with a spring constant of 0.12 N/m and a free resonance frequency of 23 kHz was employed.

Raman spectroscopy (WITec alpha300 Ra) with a 532 nm laser was used to examine the wear tracks on the steel disc. Care was taken to minimize laser damage of the tribofilm while having a reasonable signal-to-noise ratio. Raman spectra were obtained at a laser power of 10 mM, with an integration time of 0.5 s and 10 accumulations.

The chemistry of worn surfaces was obtained by X-ray photoelectron spectroscopy (XPS, Thermo Fisher K-Alpha spectrophotometer). The XPS high resolution spectra were calibrated by using C 1s as a reference at 284.8 eV.

Time-of-flight secondary ion mass spectrometry (ToF-SIMS, IONTOF, Muenster) was performed to examine the chemistry of worn surfaces. The secondary ions were generated by a bismuth primary ion beam at 25 keV. The spot size was 100 × 100 μm. The middle area and the end area on the wear track were examined. Signals that are outside of wear track were also collected and were used a reference. Both positive and negative spectra were collected for each sample.

The adsorption of OFM on an iron oxide surface was carried out on a quartz crystal microbalance with a dissipation instrument using a QSense E1 with a temperature-controlled flow cell (Biolin Scientific). A Fe_2_O_3_ coated, AT-cut quartz sensor with a fundamental frequency of 5 MHz was used. Prior to the adsorption test, the sensor and the flow cell were rinsed with isopropyl alcohol under ultrasonication for 30 min, and the sensor was then cleaned with UV ozone for another 30 min. The wetted materials in the flow path were made of PTFE (tubing), PEEK (fittings), ETFE (ferrules), Kalrez (O-ring and gasket), and titanium (flow cell). Solutions were pulled into the flow cell by an Ismatec Reglo Digital Pump with a MasterflexLive. The resonance frequencies (*f*_*n*_) and dissipations (*D*_*n*_) of the sensor for overtones *n* = 1, 3, 5, 7, 9, 11, and 13 were recorded. During an adsorption test, hexadecane was first flowed into the flow cell to obtain a baseline, followed by 1 mM OFM in hexadecane. After 3.2 h, hexadecane was pumped into the flow cell again to flush the sensor surface. All tests were carried out at 50 °C with a flow rate of 0.098 mL/min. Note that the frequency shift can be attributed to surface adsorption (Δ*f*_adsorption_), liquid loading (Δ*f*_loading_), and liquid trapping (Δ*f*_trapping_).^48,49^ In our tests, Δ*f*_loading_ and Δ*f*_trapping_ can be negligible because the introduction of low-concentration OFMs does not change the viscosity of hexadecane, and the surface roughness of the sensor is low (Ra = 0.70 nm). Since the dissipation shift resulting from the adsorbed film is very small, it suggests that the film is rigid and the adsorbed mass (Δ*m*) on Fe_2_O_3_ surface was calculated by Sauerbrey equation,^13,49^ as following:
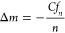
1where *C* is related to the quartz sensor properties, and it is 17.7 mg/(m^2^·Hz) in our case.^49^*n* is the harmonic overtone number, and *f*_*n*_ is the frequency shift of each overtone. As different overtones gave the same conclusion, only results from *n* = 3 was used and plotted as an adsorption curve in this manuscript. All tests were run at least twice, and all results were reproducible.

## Supporting Information Available

^1^H NMR spectra of OFMs, the tribological performance of OFMs at different temperatures, additional optical images of wear track, AFM topography image of different wear track, surface chemistry analysis on wear track, QCM-D adsorption test of OFMs, and flash-temperature calculation methods (PDF)
