# Supplementary material for: Position of Carbonyl Group Affects Tribological Performance of Ester Friction Modifiers
Source: ACS Appl Mater Interfaces. 2024 Mar 8;16(11):14252–62. doi: 10.1021/acsami.3c16432 (PMC10958443; doi:10.1021/acsami.3c16432)
Supplement: Supplementary file 1 — am3c16432_si_001.pdf [file am3c16432_si_001.pdf]

Supporting Information

Position of Carbonyl Group Affects Tribological Performance of Ester Friction Modifiers

Wei Song[1], Sophie Campen[1], Huw Shiel[2], Chiara Gattinoni[3], Jie Zhang[1], and Janet S. S. Wong[1,*]

[1]The Tribology Group, Department of Mechanical Engineering, Imperial College London, Exhibition Road, South Kensington, London, SW7 2AZ, UK

[2]Department of Material Science, Imperial College London, Exhibition Road, South Kensington, London, SW7 2AZ, UK

[3]Department of Chemical and Energy Engineering, London South Bank University, 103 Borough Rd, London SE1 0AA, United Kingdom

*j.wong@imperial.ac.uk

Supplementary Note 1. The ^{1}H and ^{13}C NMR spectra of OG and SG

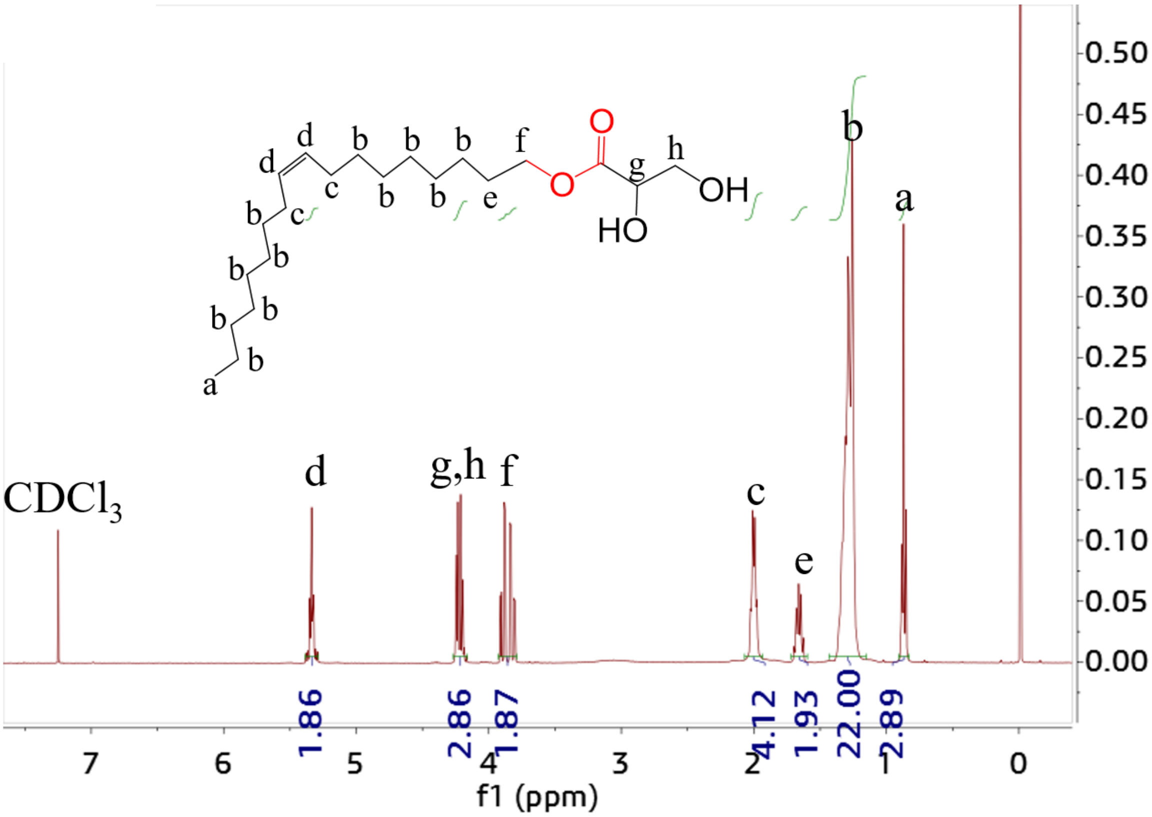

Figure S1 The ^{1}H NMR spectrum of OG

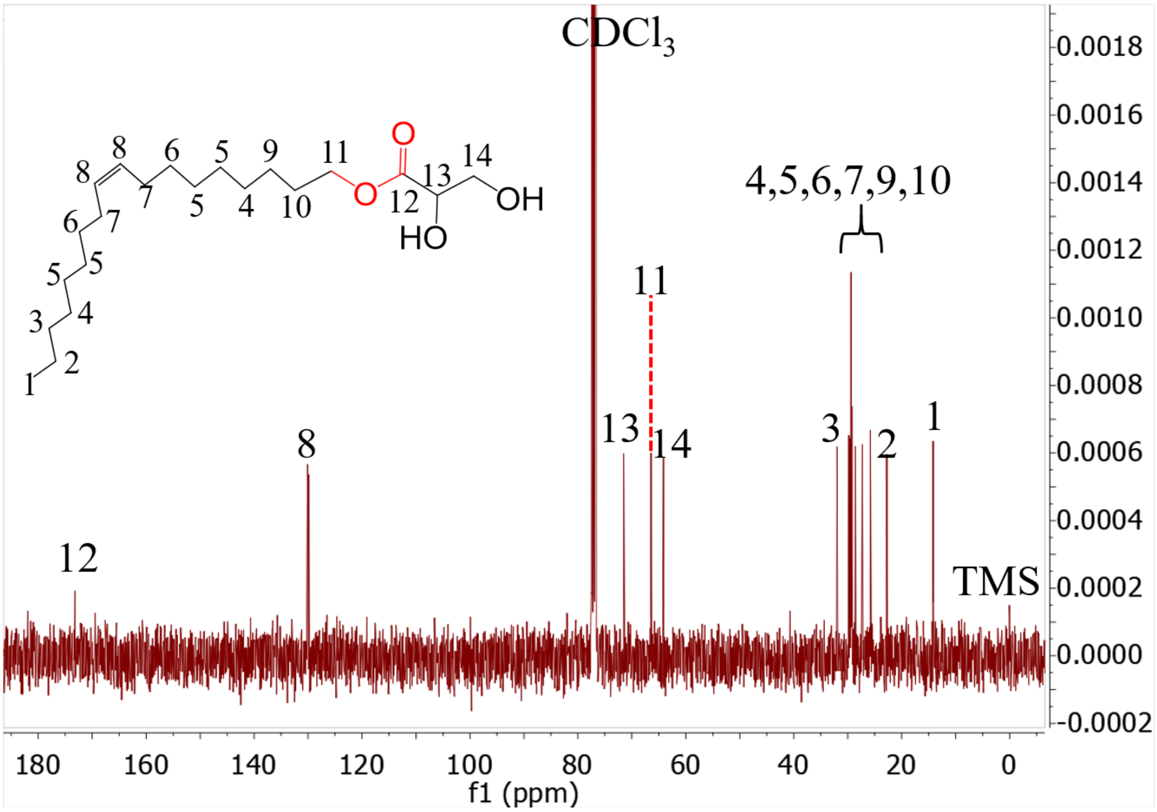

Figure S2 The ^{13}C NMR spectrum of OG

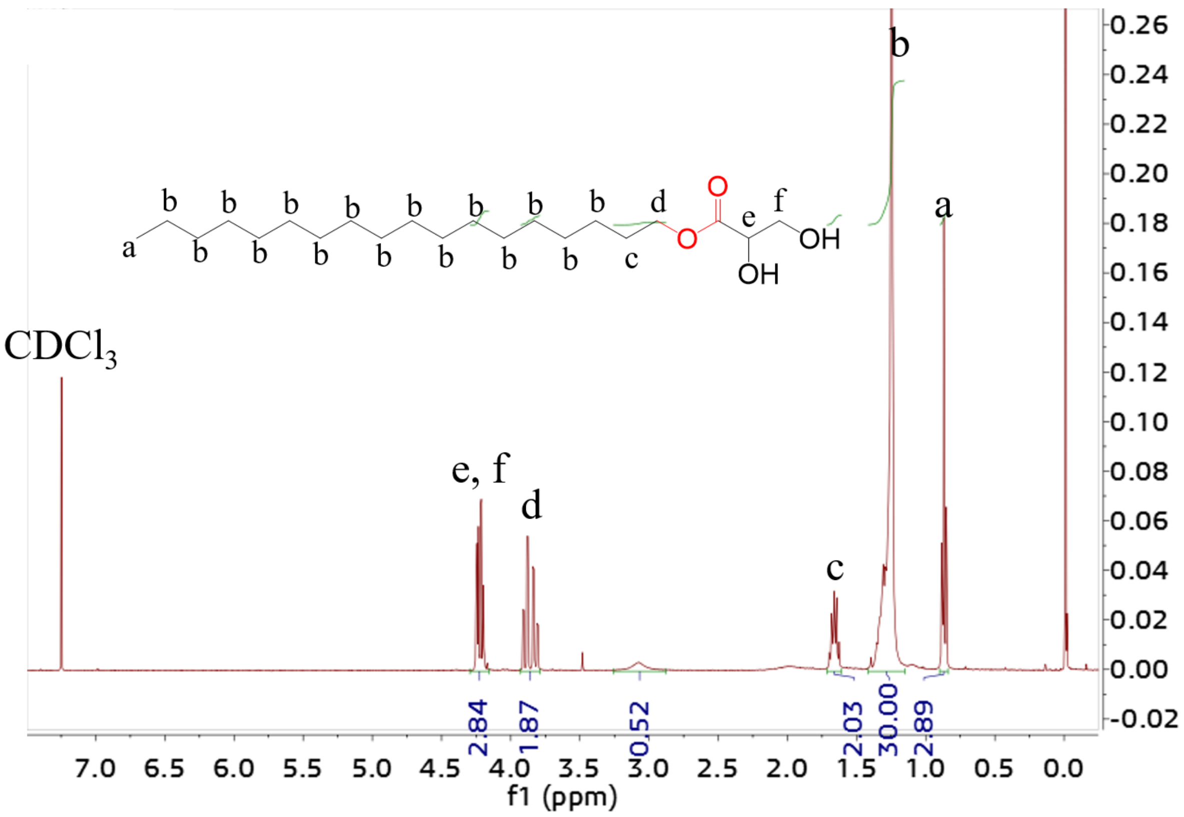

Figure S3 The ^{1}H NMR spectrum of SG

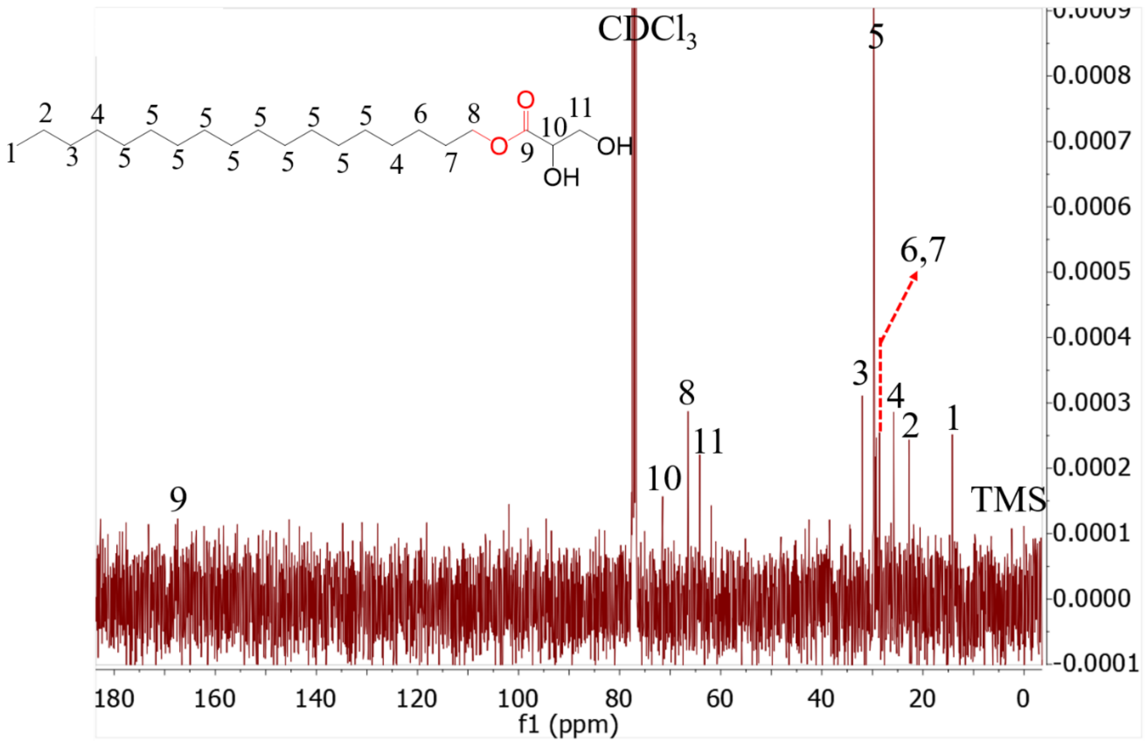

Figure S4 The ^{13}C NMR spectrum of SG

Supplementary Note 2. The tribological performance of OG, GMO at different temperature

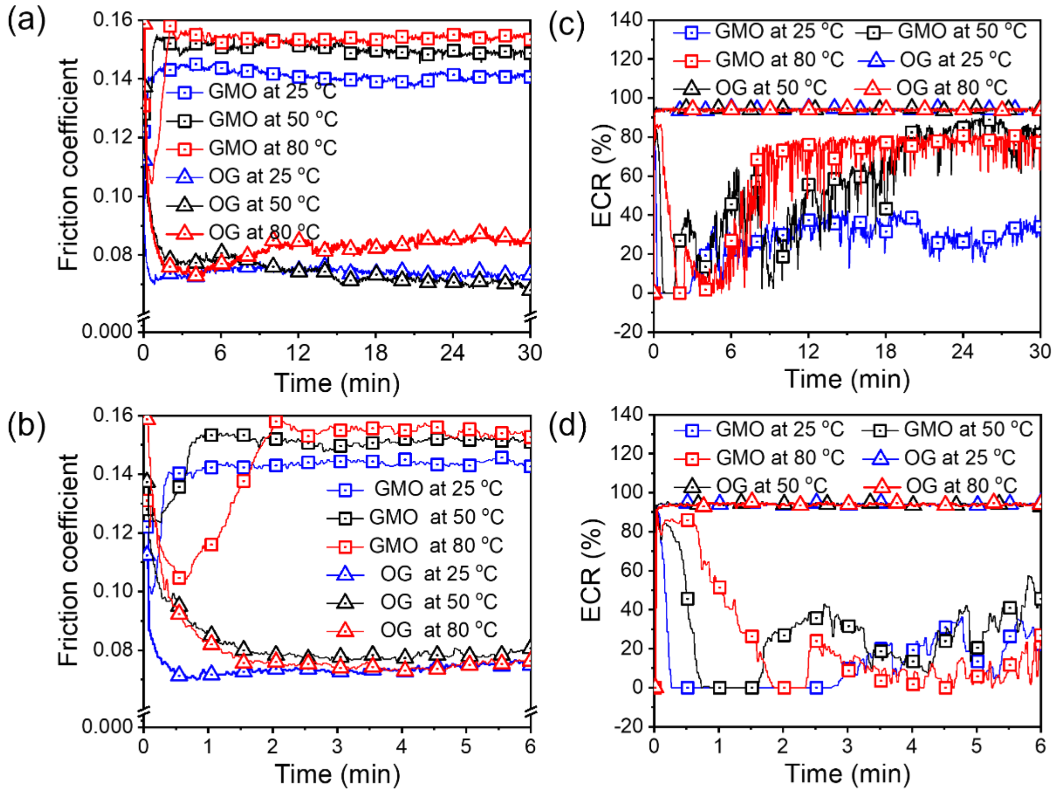

Figure S5 (a) The friction coefficient, and (c) ECR of 1 mM GMO and 1 mM OG at different temperature. (b)(d) are the first 6 min of (a)(c).

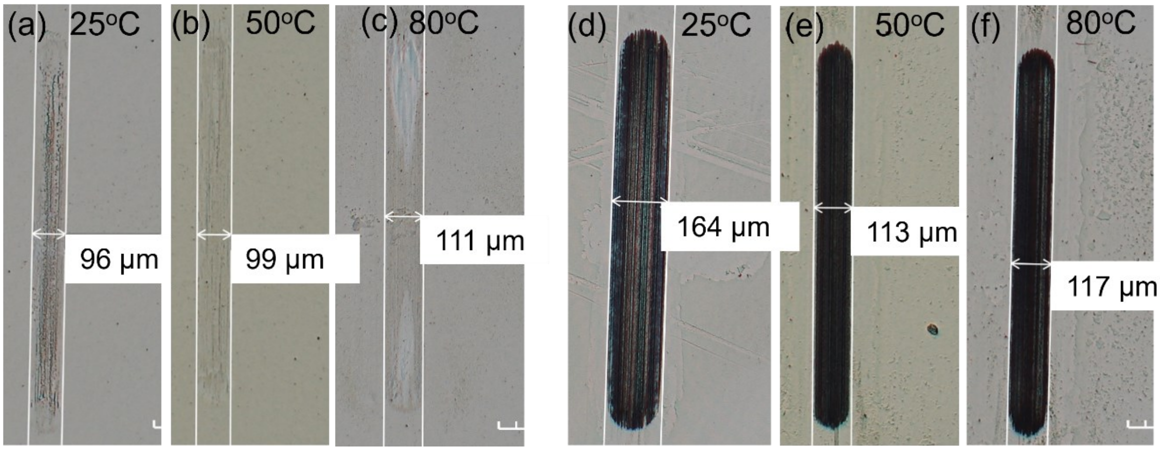

Figure S6 The optical images of wear track formed in 1 mM OG at (a) 25 °C, (b) 50 °C, (c) 80 °C, and 1 mM GMO at (d) 25 °C, (e) 50 °C, (f) 80 °C.

Supplementary Note 3. The tribological performance of SG, GMS at different temperature

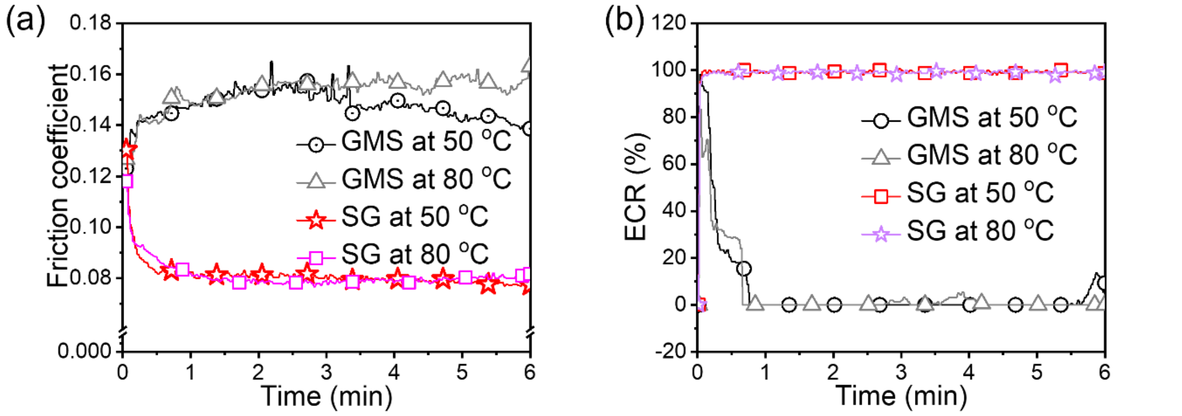

Figure S7 (a) The friction coefficient, and (b) ECR of 1 mM GMS and 1 mM SG at different temperature for the first 6 mins.

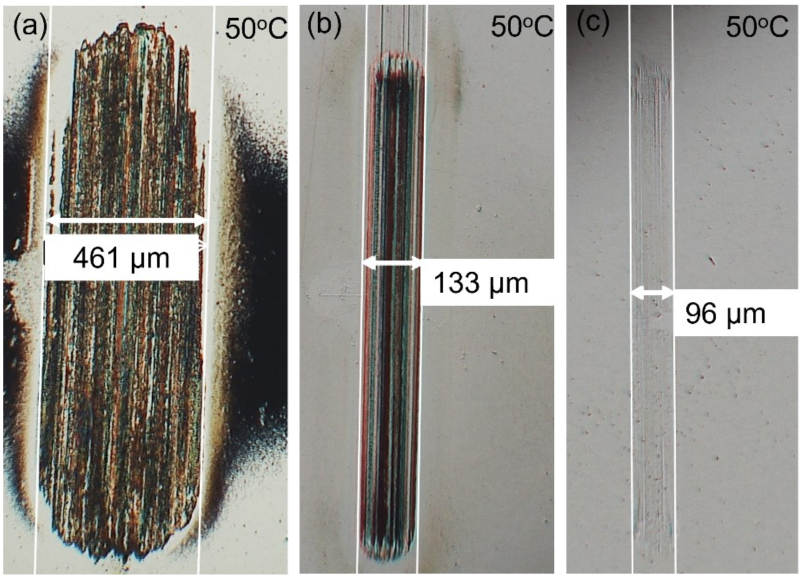

Figure S8 The optical images of wear track formed at 50 °C, given by (a) hexadecane, (b) 1 mM GMS, (c)1 mM SG

Supplementary Note 4. The optical images of wear track before and after hexane rinsing

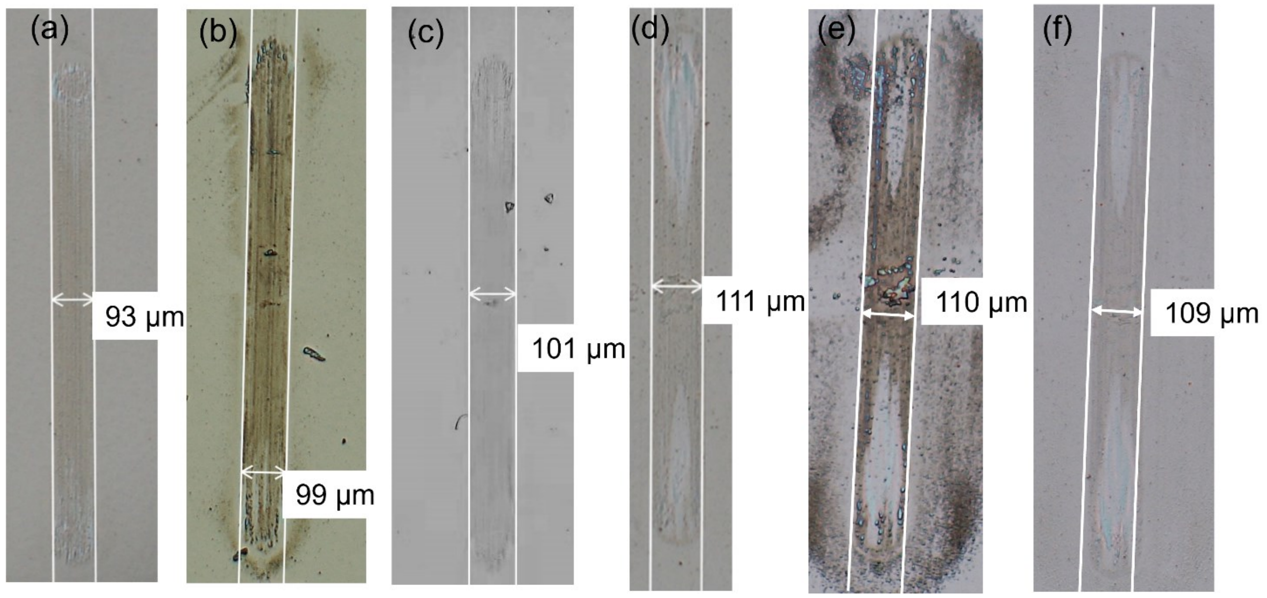

Figure S9 The wear track formed in (a)(b)(c) 1 mM SG at 80 ºC, and (d)(e) (f) 1 mM OG tested at 80 ºC. (a)(d) are before hexane rinsing, (b)(e) are after hexane rinsing, (c)(f) are immersed in hexadecane after hexane rinsing.

Supplementary Note 5. The average friction coefficient of OFM at different temperatures

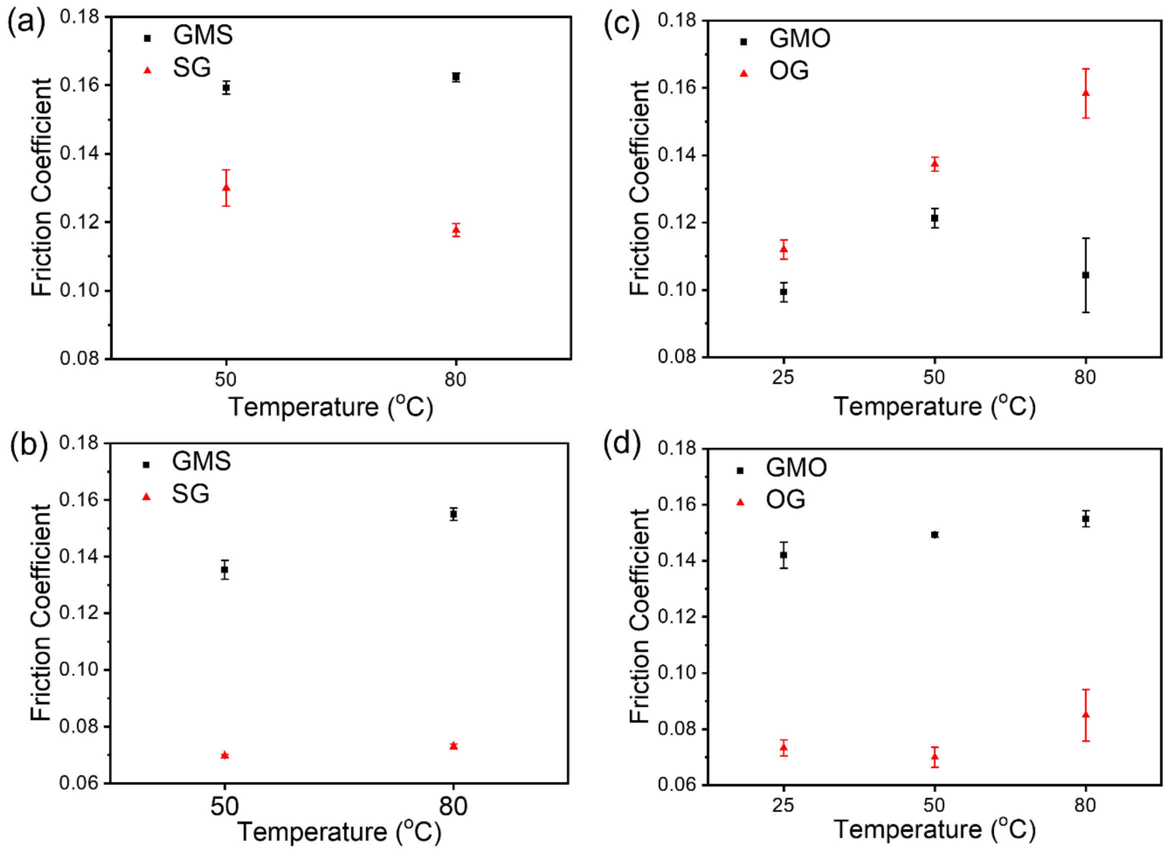

Figure S10 The (a)(c) maximum, and (b)(d) steady friction coefficient of OFM

Supplementary Note 6. Effect of Concentration on friction coefficient of OG and GMO

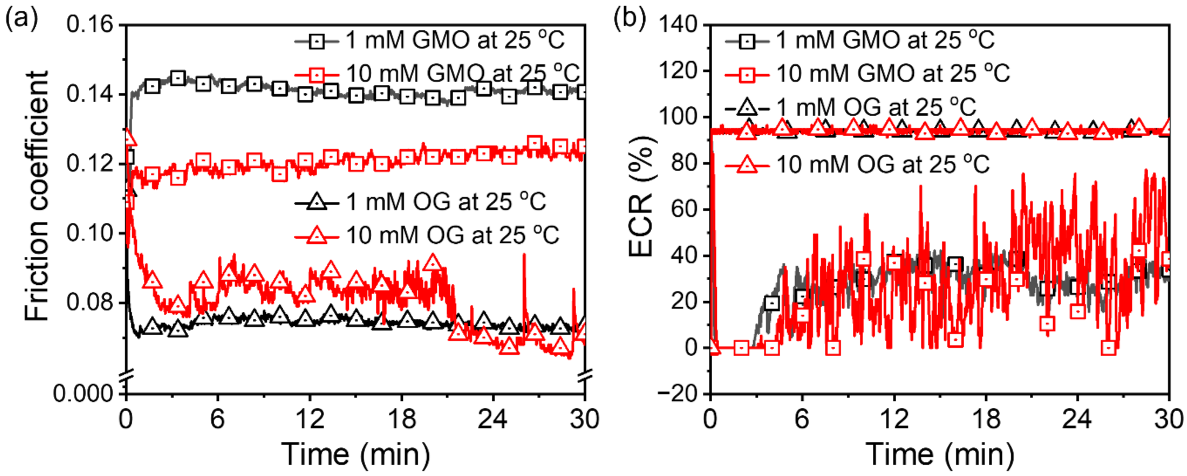

Figure S11 (a) Friction coefficient, and (b) ECR of OFMs with different concentrations.

Supplementary Note 7. The stability of the glycerate tribofilm in air

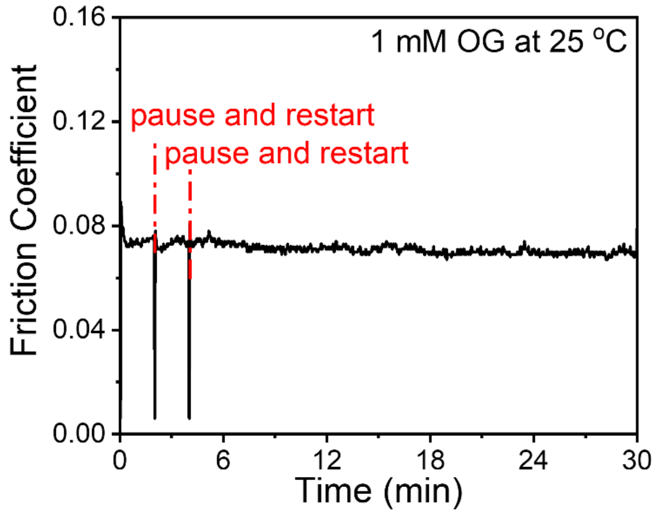

Figure S12 (a)(c) The friction coefficient of 1 mM OG at 25 ºC, which is interrupted at 2 min and 4 min.

Supplementary Note 8. The surface morphology of unrubbed steel and of wear tracks lubricated with additives

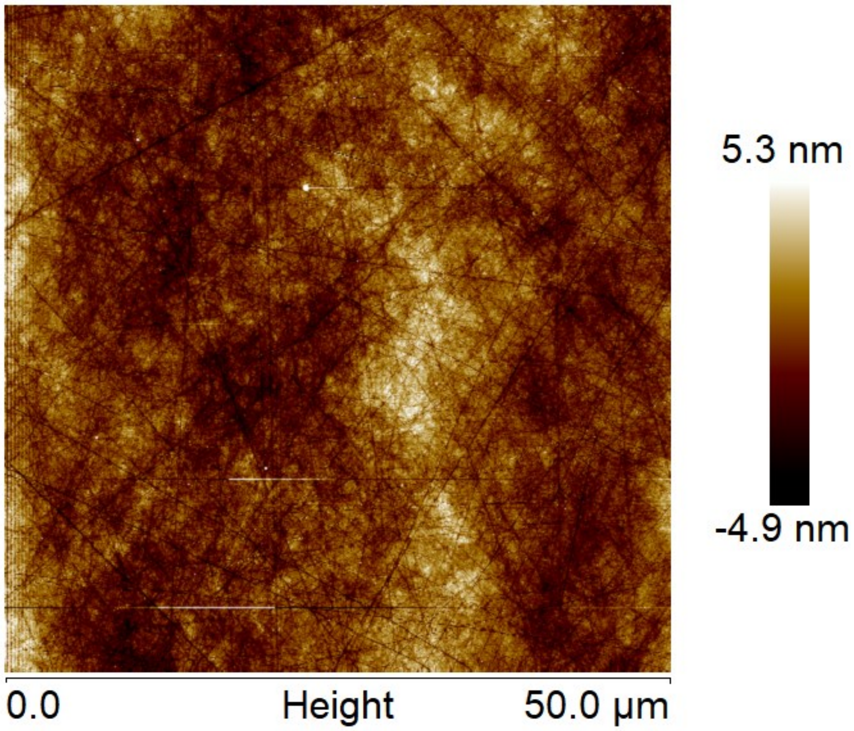

Figure S13 AFM morphology of unrubbed steel disc in hexadecane.

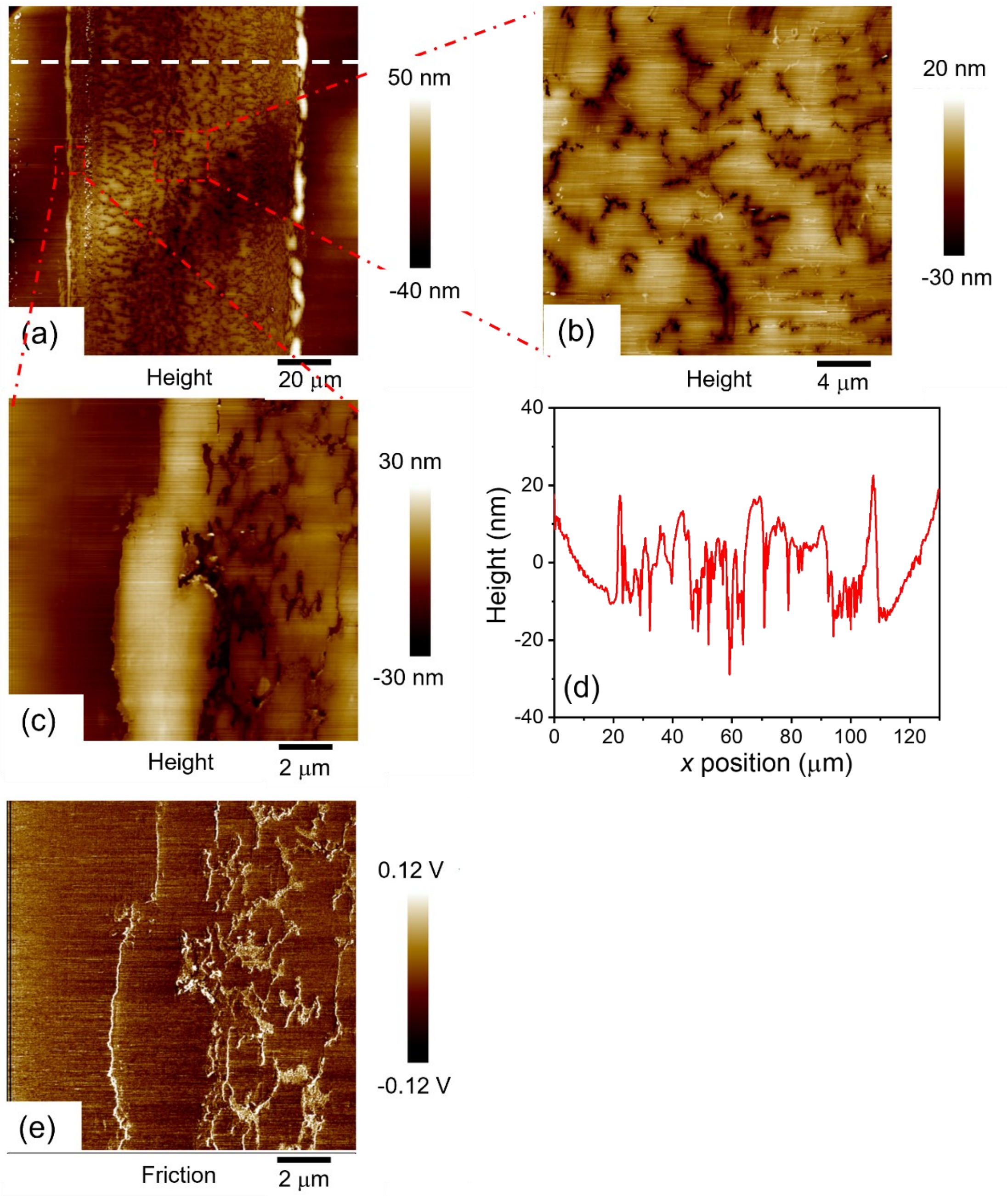

Figure S14 Morphology of steel disc wear track formed in 1 mM OG at 80 ºC. (a)(b)(c) AFM height image of the wear track. (b)(c) are magnified regions in the centre and edge of the wear track, see red box in (a). (d) The height profiles of the white lines in (a). (e) The corresponding lateral force image of (c).

`

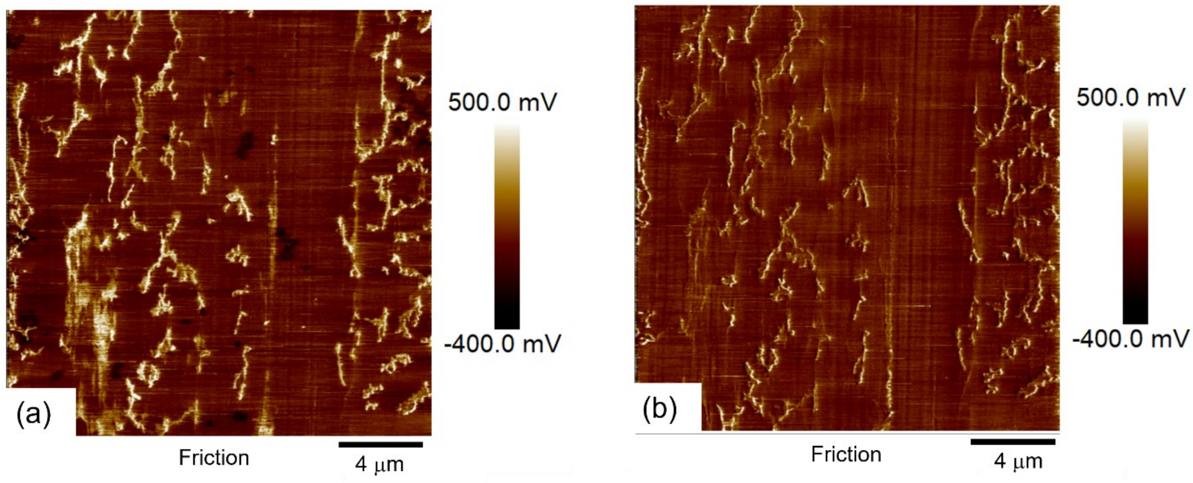

Figure S15 The corresponding lateral force images of (a) Figure 2f, (b) Figure 2g.

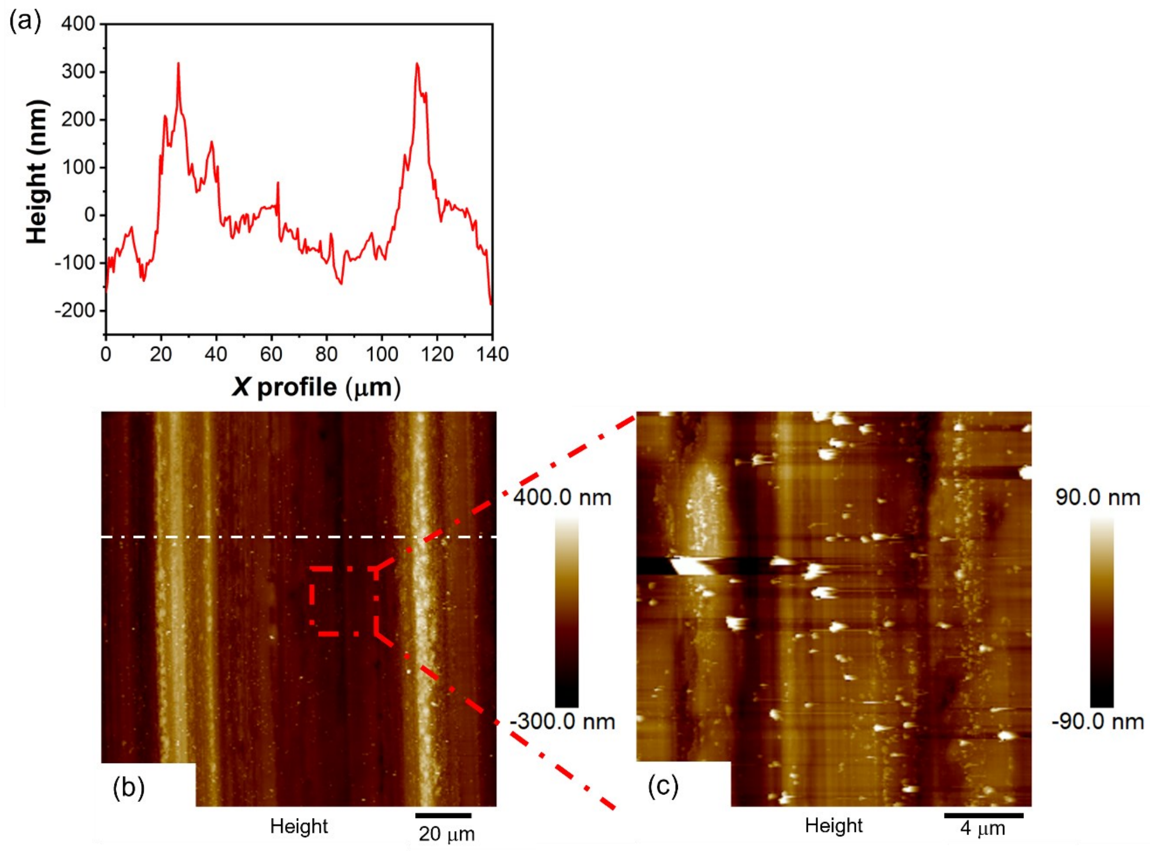

Figure S16 (a)The height profile of the white line in (b). (b) (c) The AFM height images of the wear track centre formed in 1 mM GMS at 80 ºC. (c) is magnified image of a region in the centre of the wear track, see red box in (b).

Supplementary Note 9. The chemical analysis of wear tracks

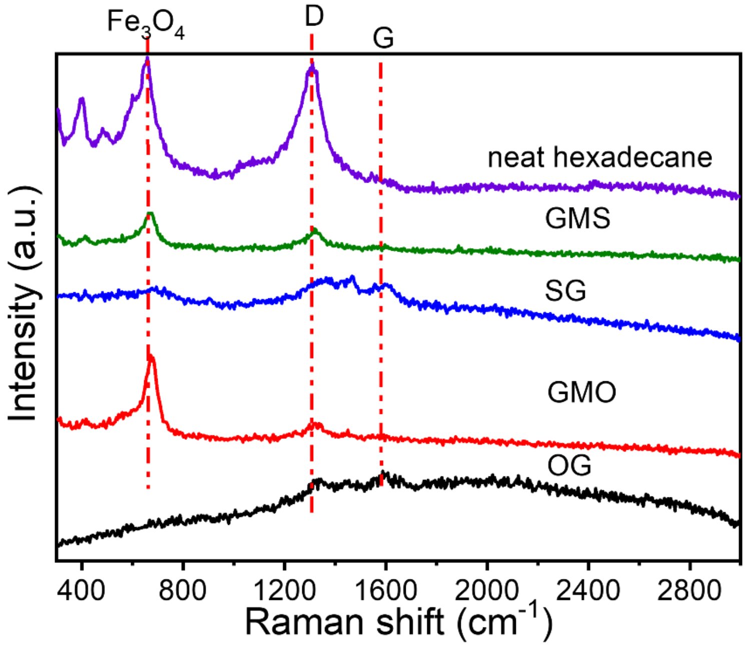

Figure S17 Raman spectra before normalisation of wear tracks formed at 80 °C in hexadecane with and without 1 mM additives.

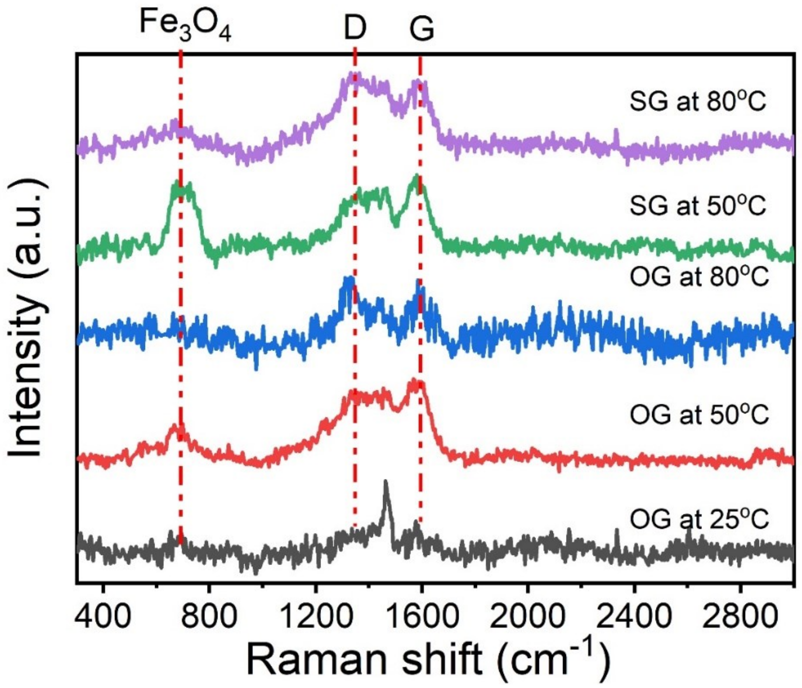

Figure S18 Normalised Raman spectra of wear tracks formed in hexadecane with and without 1 mM additives at different temperatures. The intensity of the strongest peak of each spectrum is set at 1.

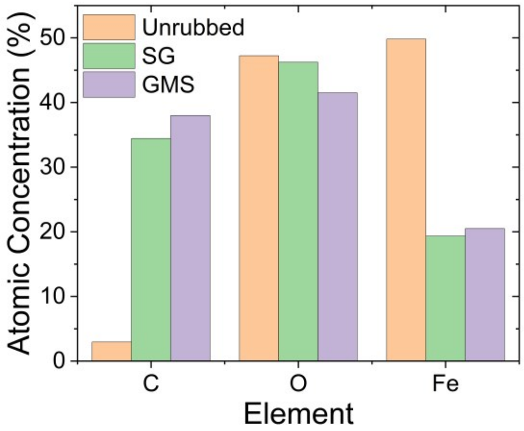

Figure S19 Concentration of C, O and Fe on worn surfaces.

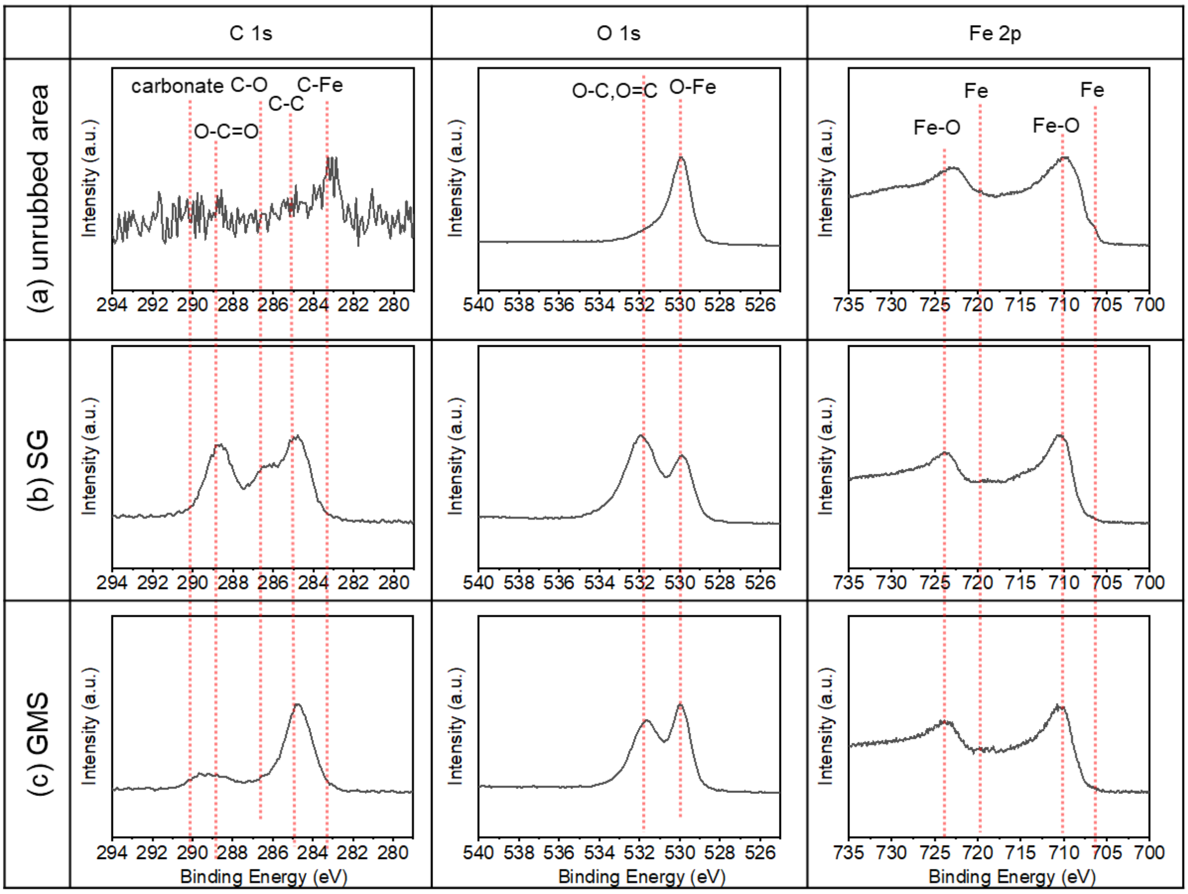

Figure 20 Normalised C1s, O1s and Fe 2p XPS high resolution spectra before normalisation of (a) unrubbed area, (b) 1 mM SG lubricated surface and (c) 1 mM GMS lubricated surface.

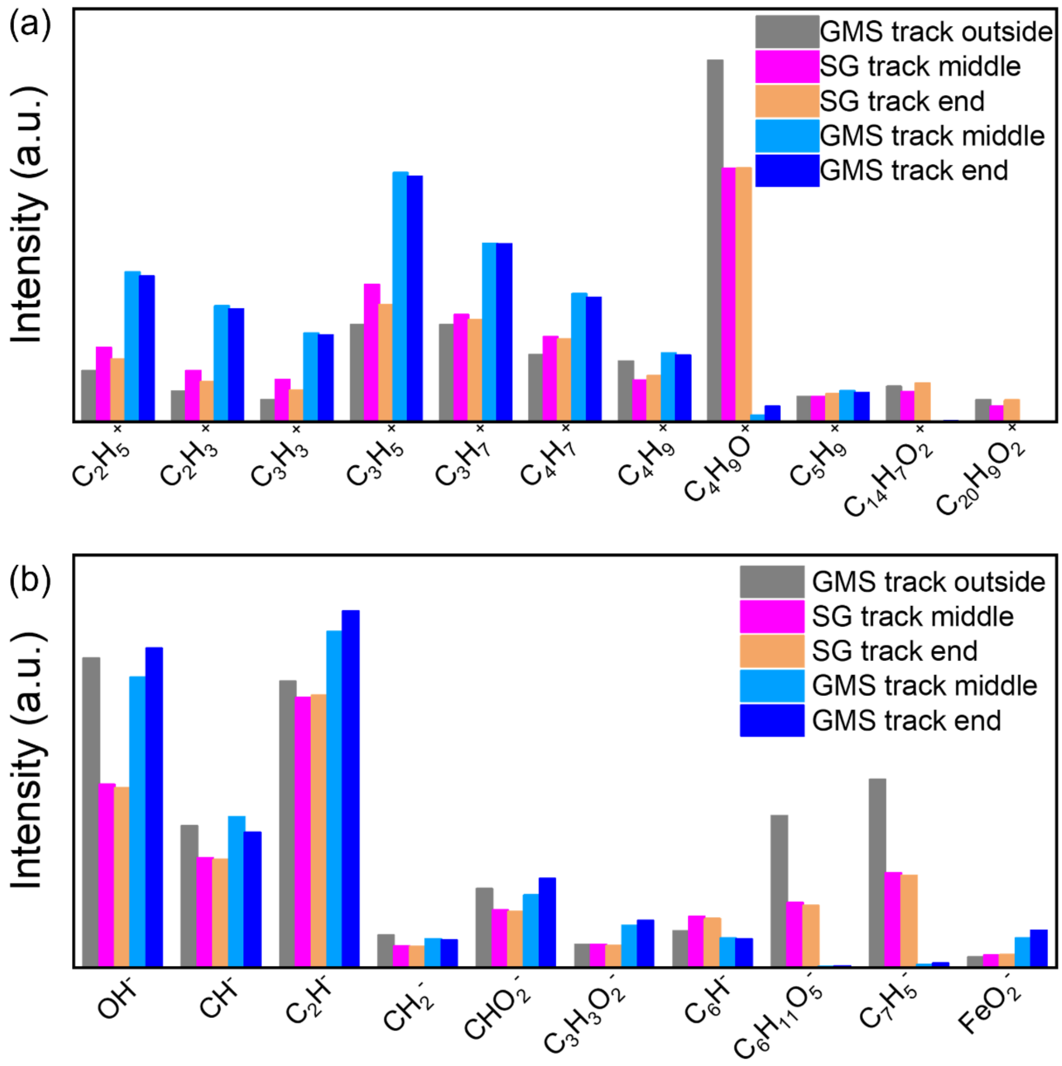

Figure S21 The ion fragment intensity on unrubbed area, 1 mM SG lubricated surface and 1 mM GMS lubricated surface, determined by ToF-SIMS under (a) positive mode, and (b) negative mode

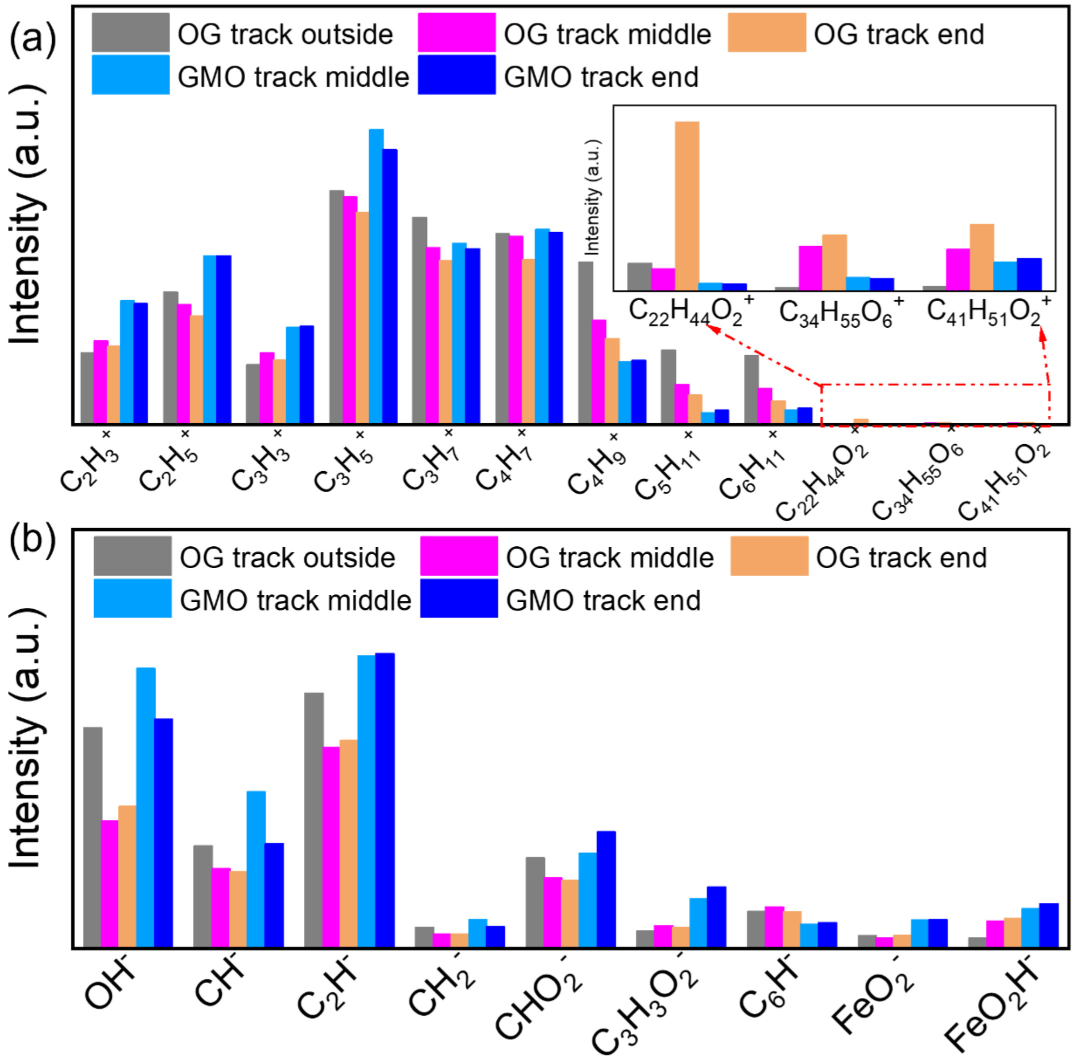

Figure S22 The ion fragment intensity on unrubbed area, 1 mM OG lubricated surface and 1 mM GMO lubricated surface, determined by ToF-SIMS under (a) positive mode, (b) negative mode.

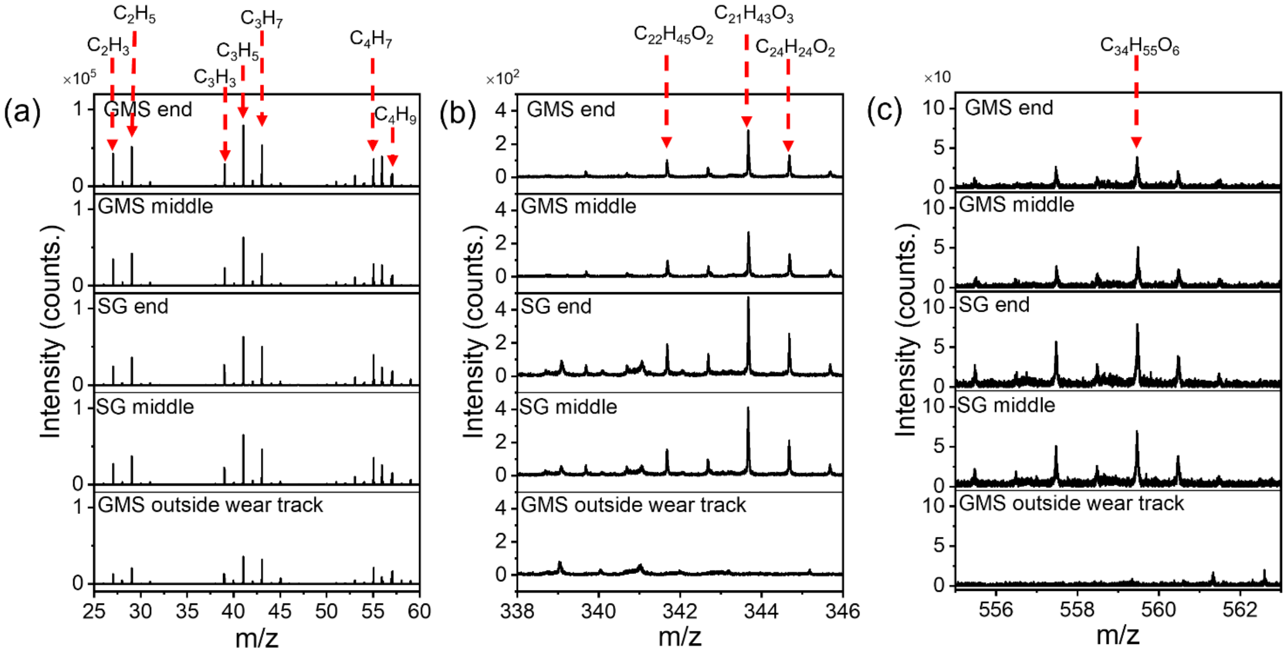

Figure S23 The mass spectra on wear track formed by 1 mM GMS and 1 mM SG. (a) m/z: from 25 to 60; (b) m/z: from 338 to 346; (c) m/z: from 555 to 563.

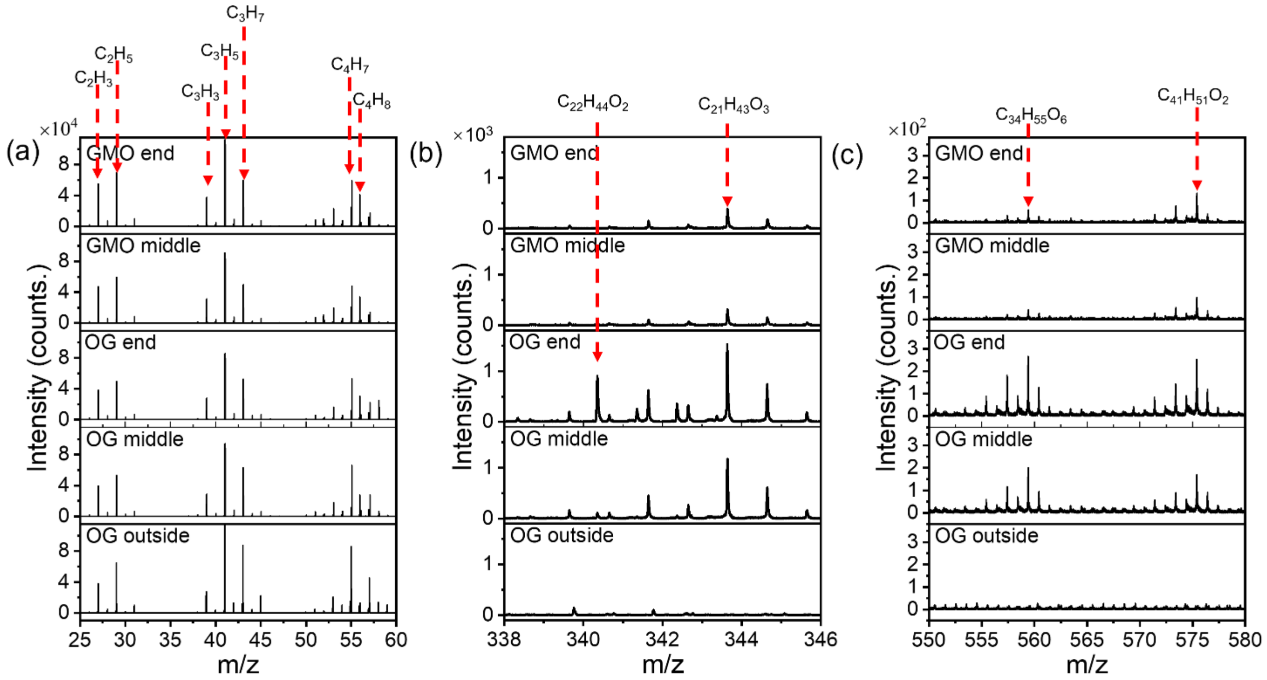

Figure S24 The mass spectra on wear track formed by 1 mM GMO and 1 mM OG. (a) m/z: from 25 to 60; (b) m/z: from 338 to 346; (c) m/z: from 550 to 580.

Supplementary Note 10 Tribological Performance of 1 mM SG under different load and with different durations.

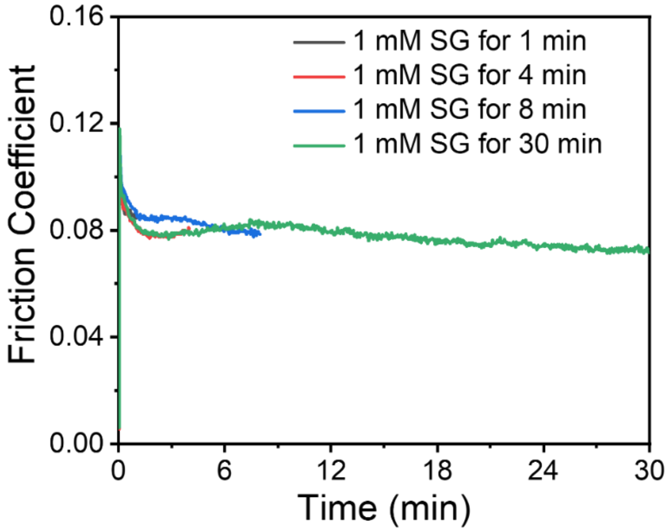

Figure S25 The friction coefficient of 1 mM SG in hexadecane at 80 ºC under 5N for different time

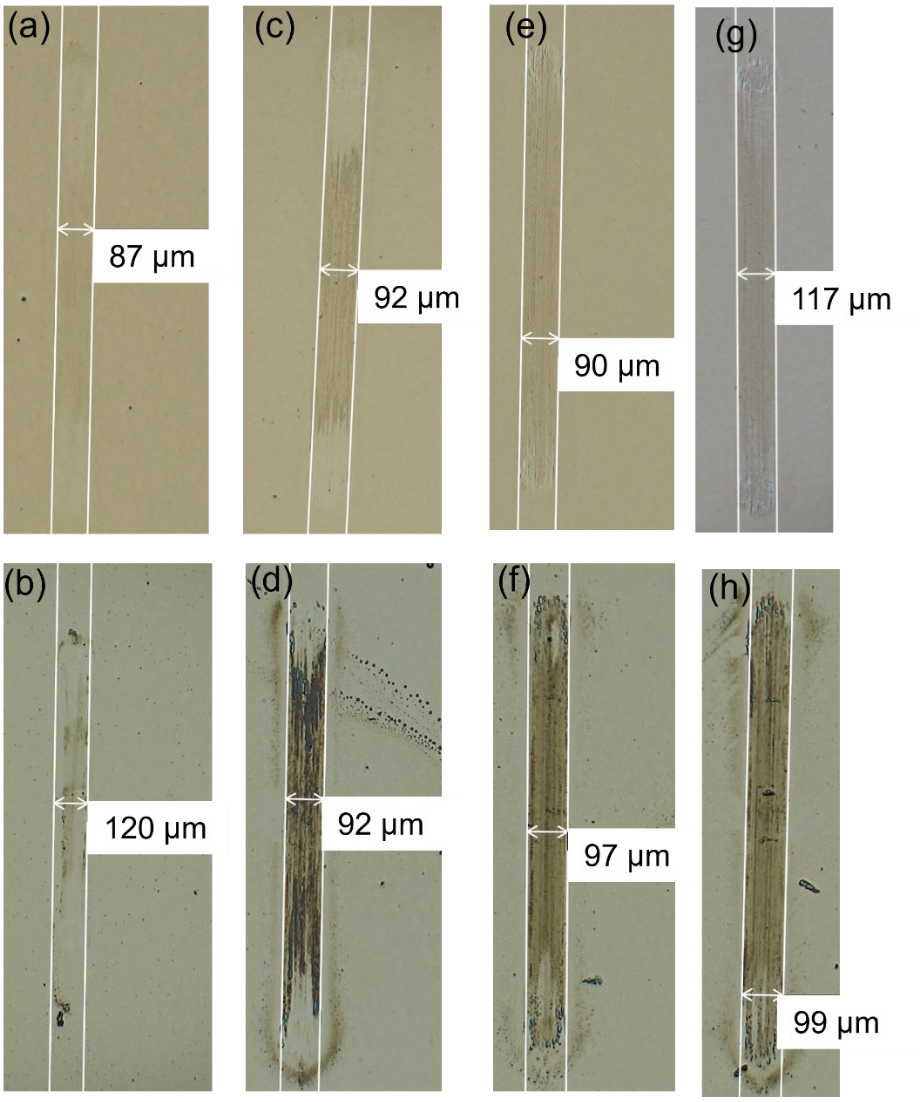

Figure S26 The wear tracks formed by 1 mM SG at 80 ºC under 5N for (a)(b) 1 min, (c)(d) 4min, (e)(f) 8 min, (g)(h) 30 min. (a)(c)(e)(g) are before hexane rinsing, (b)(d)(f)(h) are after hexane rinsing.

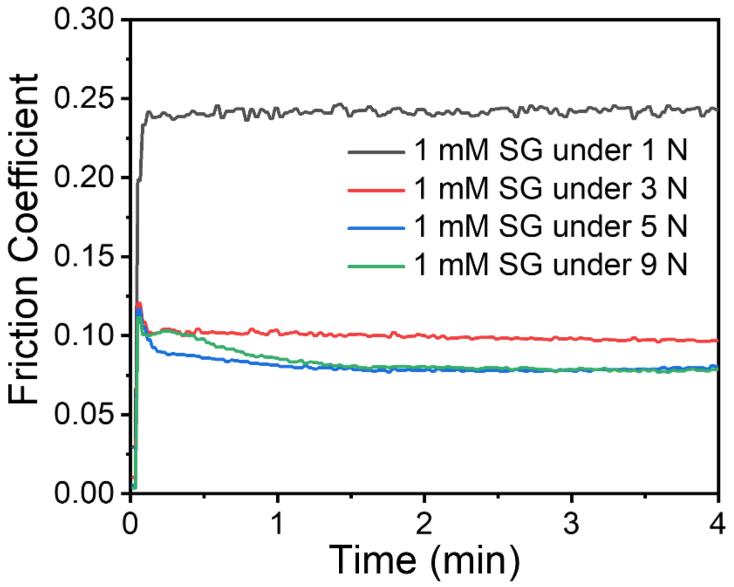

Figure S27 The friction coefficient of 1 mM SG in hexadecane at 80 °C under different load for 4 min.

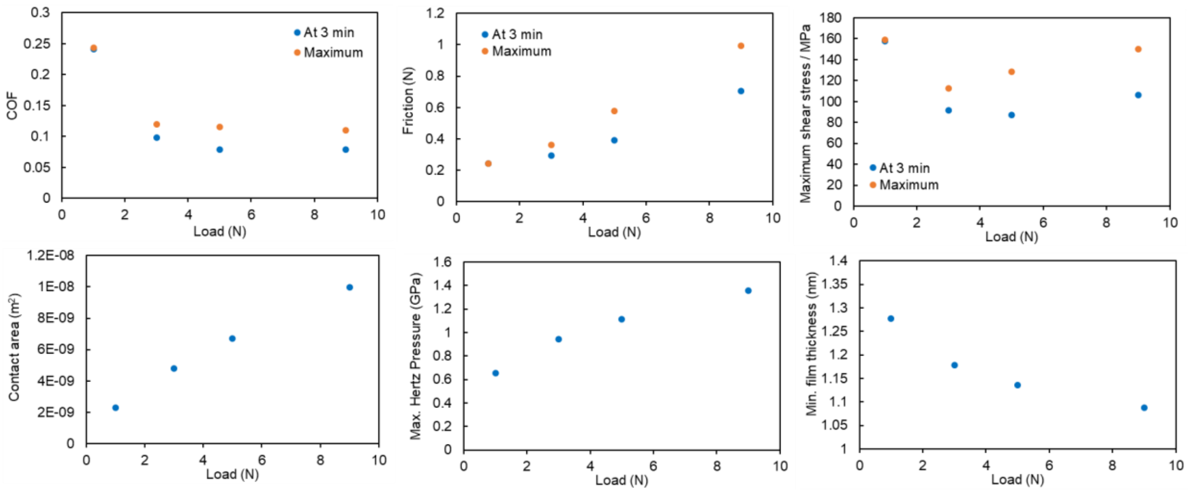

Figure S28 Estimated contact shear and associated calculation for the friction tests shown in Figure S27. was calculated using maximum friction coefficient and Hertzian contact with steady state friction coefficient and area of wear scar of the balls

Supplementary Note 11 The tribological performance and surface analysis of 1 mM SG under 1 N for 10 h

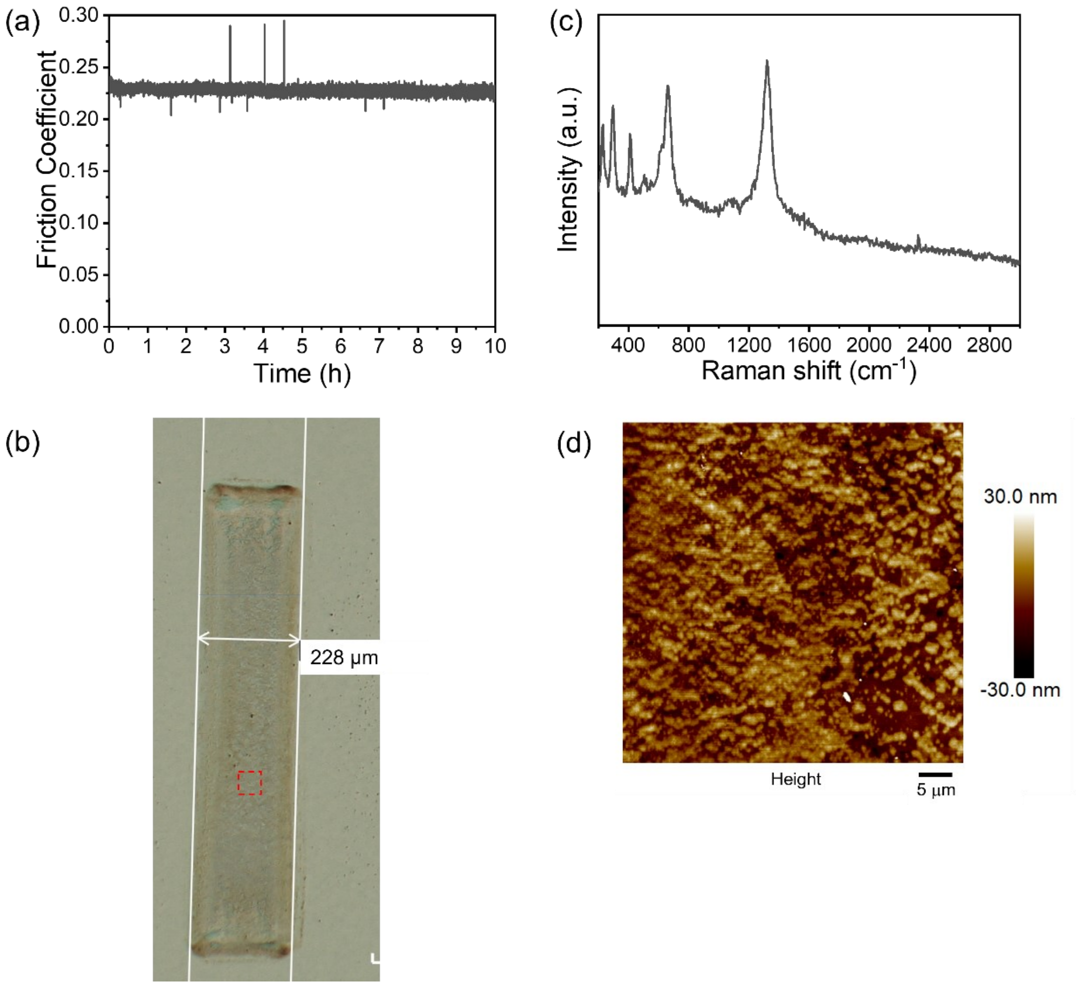

Figure S29 (a) The friction coefficient, (b) wear track image of 1 mM SG lubrication under 1 N at 80 °C for 10 h. (c) The Raman spectrum, and (d) AFM height image of the red box in (b).

Supplementary Note 12. The surface morphology of wear track formed by 1 mM SG under different load for 4 min.

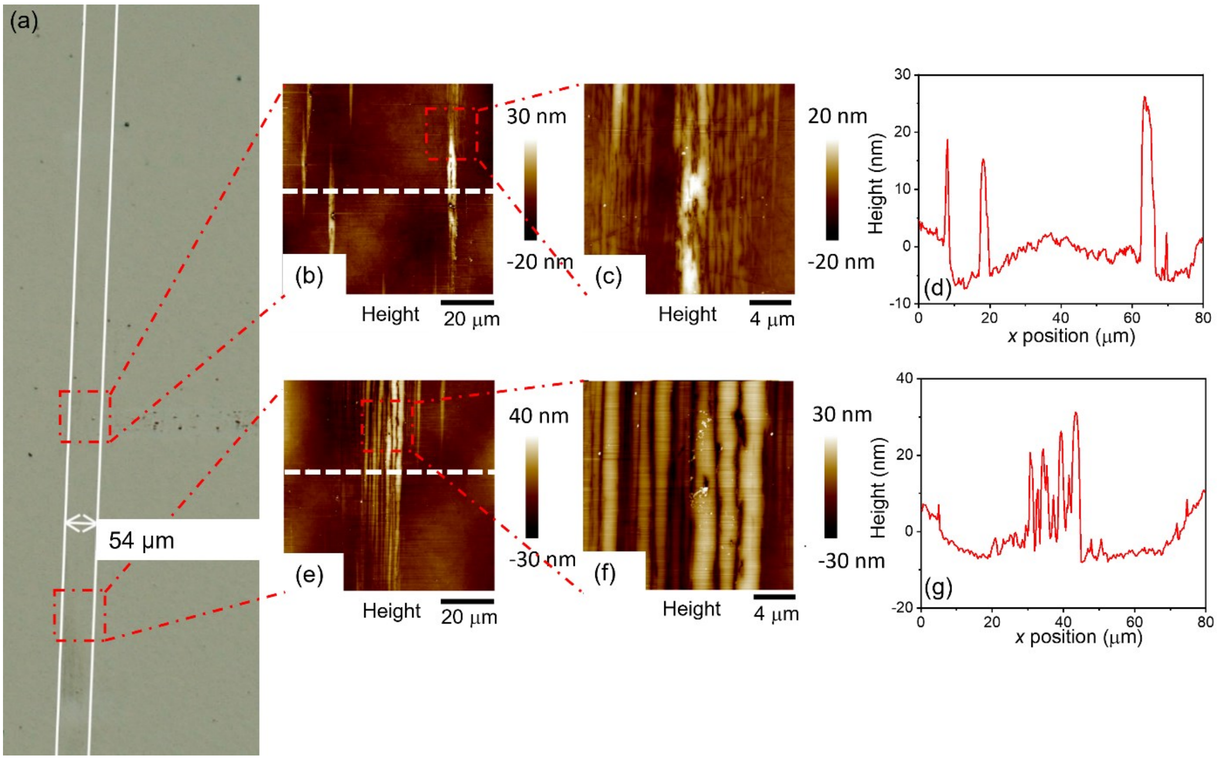

Figure S30 Morphology of wear scars formed in 1 mM SG under 1 N for 4 min. AFM height image of (b)(c) middle region, and (e)(f) transition region of wear track. (d)(g) are the height profiles of the white line in (b)(e.)

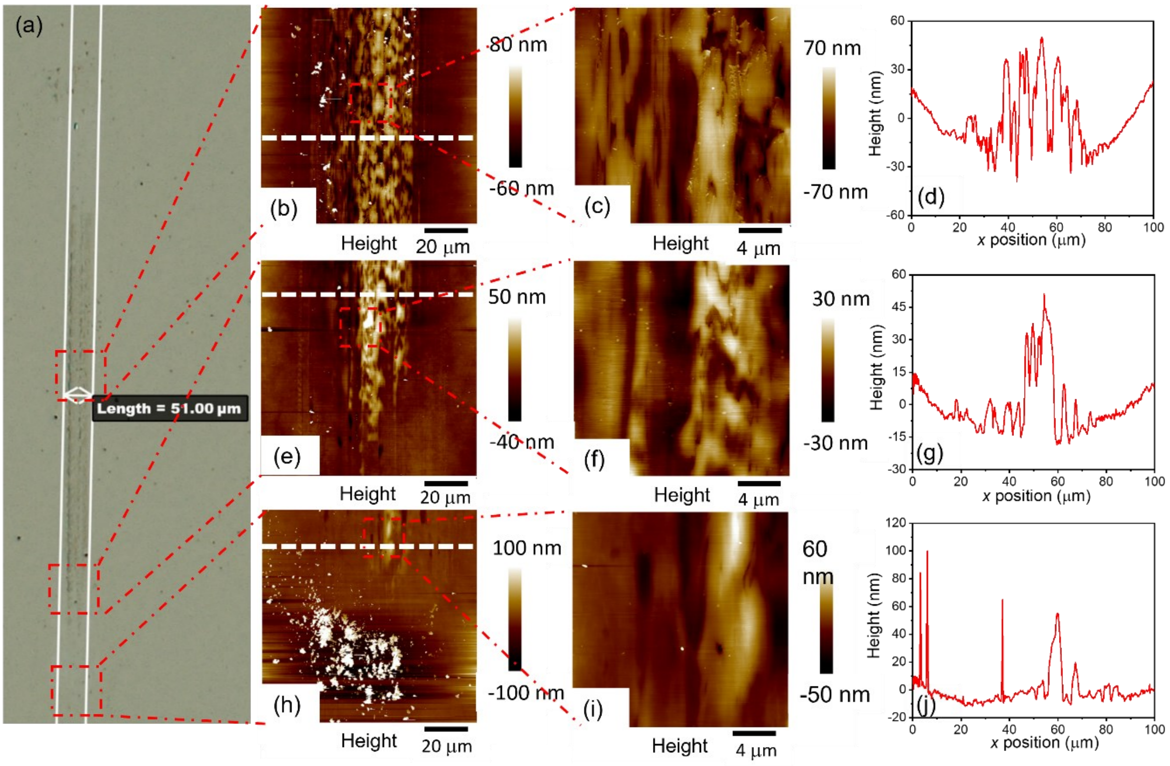

Figure S31 Morphology of wear scars formed in 1 mM SG under 3 N for 4 min. AFM height image of (b)(c) middle region, (e)(f) transition region, and (h)(i) end region of wear track. (d)(g) (j) are the height profiles of the white line in (b)(e)(h).

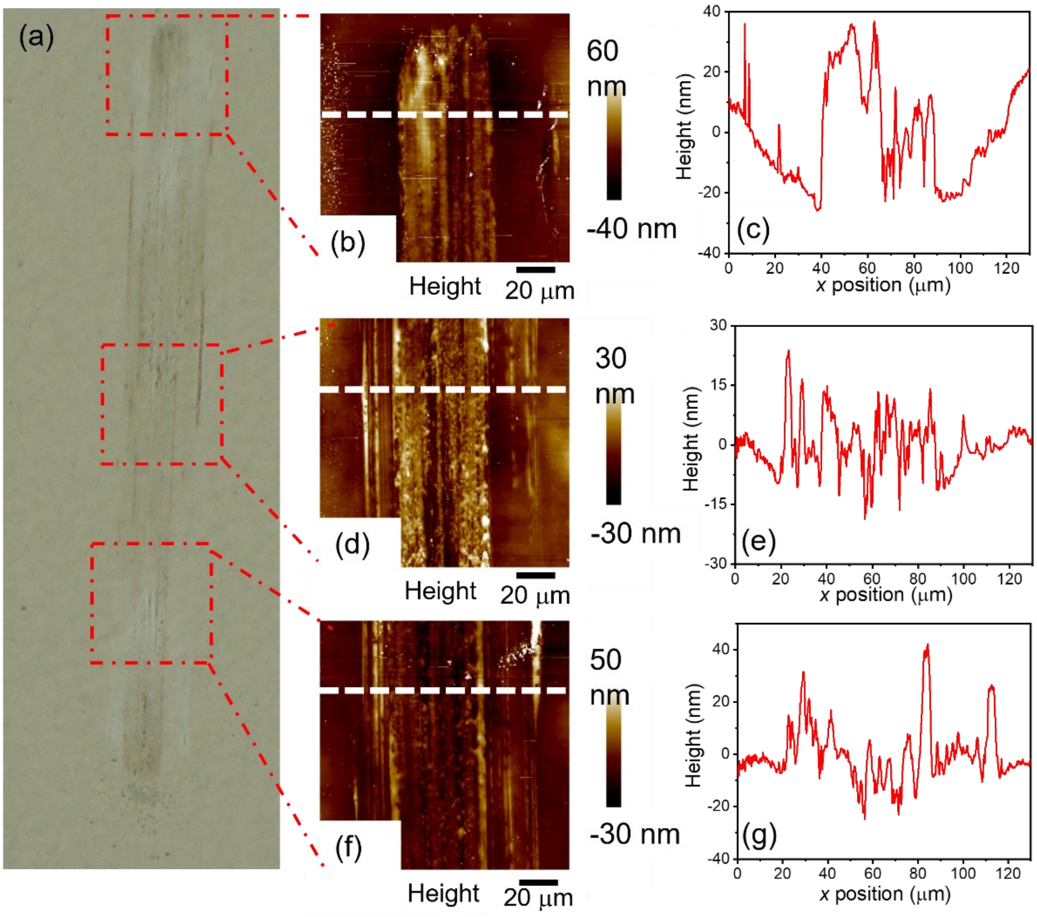

Figure S32 Morphology of wear scars formed in 1 mM SG under 9 N for 4 min. AFM height image of (b) end region, (d) middle region, and (f) transition region of wear track. (c)(e) (g) are the height profiles of the white line in (b)(d)(f).

Supplementary Note 13. The adsorption of additives on steel surface

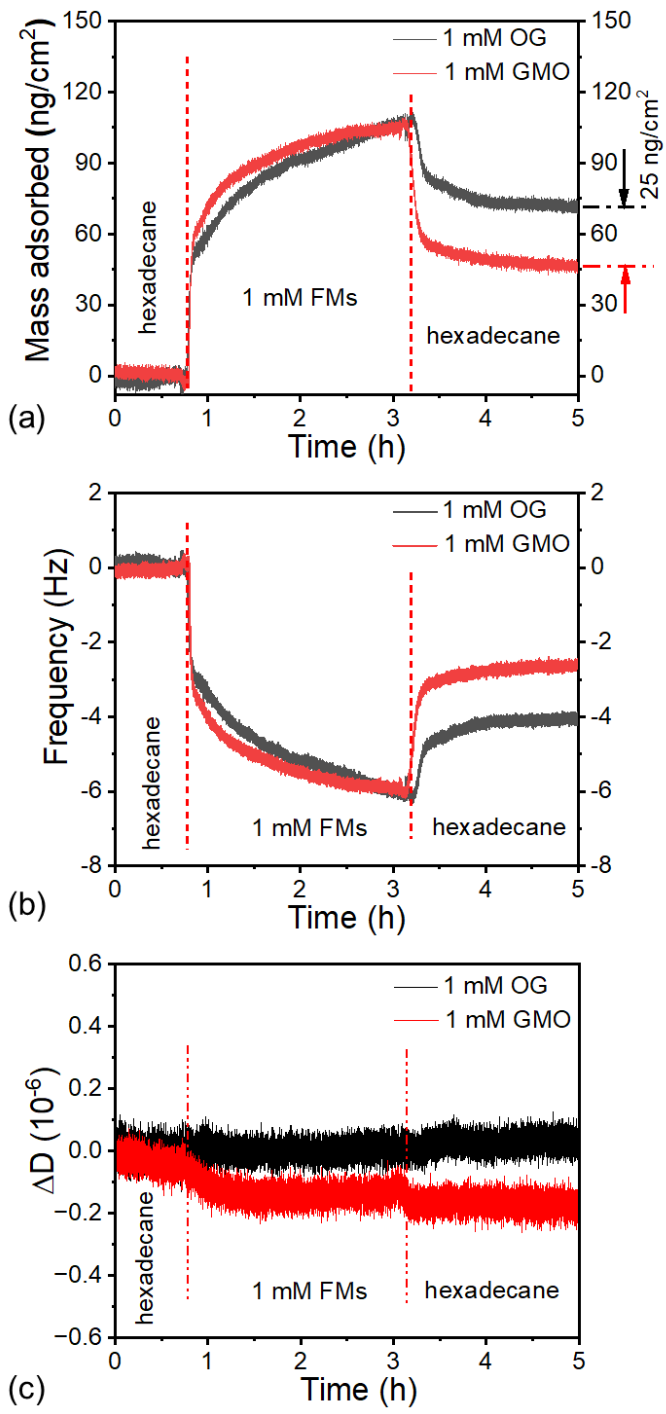

Figure S33 QCM-D results of 1 mM OG/GMO on steel surface based on the 3rd overtone. (a) Adsorbed mass, (b) frequency shift, (c) dissipation shift. The solution flow sequence: hexadecane to create a baseline → 1 mM FMs in hexadecane to allow a dynamic equilibrium adsorbed film to be formed → hexadecane flushing, where excess adsorbed mass are removed and only those strongly adsorbed molecules are remained.

Supplementary Note 14 The flash temperature calculation.

The flash temperature in the contact area was calculated according to Jaegers model. The following equation and the corresponding parameter in Table S15-1 were used. The calculated result was in Table S15-2

$$T_f = \frac{0.903\dot{q}a}{1.064K_{ball}+0.849K_{disc}\sqrt{Pe}} \text{ and } \alpha = \frac{0.849K_{disc}\sqrt{Pe}}{1.064K_{ball}+0.849K_{disc}\sqrt{Pe}}\pi r^2$$

Definitions for variables used in calculations and discussion of the flash temperature and temperature measurements are given below:

Tf Average flash temperature rise [ºC]

α Heat partition coefficient

μ Coefficient of friction

W Normal Load [N]

U Relative sliding velocity [m/s]

a Contact radius [m]

$A = \pi a^2$ Contact area

$p = W/A$ Average contact pressure [Pa]

$\dot{q} = \mu pU$ Heat flux per unit area [$W/m2$]

K Thermal conductivity [W/mK]

ρ Density [$kg/m3$]

σ Specific heat [J/kgK]

$\chi = K/\rho\sigma$ Thermal diffusivity [$m2 /s$]

$Pe = 2Ua/\chi$ Peclet number

Table S1 Material property parameters for flash temperature calculations

Materials	χ [m^2/s]	K [W/mK]	ρ [kg/m^3]	σ [J/kgK]
steel	0.000013	46	7700	461

Table S2 The calculated flash temperature under 5 N for different additives based on steady friction coefficient

	Flash temperature [^{o}C]
1 mM SG/OG	4.7
1 mM GMO/GMS	8.8
